# CAR19 Tregs treat murine chronic graft-versus-host disease through immune suppression without measurable B cell cytolysis

**DOI:** 10.1172/jci.insight.196197

**Published:** 2026-07-14

**Authors:** Sujeong Jin, Michael C. Zaiken, Cameron McDonald-Hyman, Christina R. Hartigan, Sara Bolivar-Wagers, Jemma H. Larson, Yiyun Peng, Sophia Hani, Megan Riddle, Asim Saha, Angela Panoskaltsis-Mortari, Eun Ko, Yujie Zhao, Rocio Amaro Marquez, Pooja Shree Marri Baskar, Cindy R. Eide, William J. Murphy, Keli L. Hippen, Geoffrey R. Hill, Jakub Tolar, Peter T. Sage, Christopher A. Pennell, Leslie S. Kean, Bruce R. Blazar

**Affiliations:** 1 Department of Pediatrics, Division of Pediatric Blood and Marrow Transplantation & Cellular Therapy, and; 2 Department of Medicine, Division of Hematology, Oncology and Transplantation, University of Minnesota Medical School, Minneapolis, Minnesota, USA.; 3 Department of Dermatology and; 4 Department of Internal Medicine, Division of Hematology and Oncology, University of California, Davis, School of Medicine, Sacramento, California, USA.; 5 Translational Science and Therapeutics Division, Fred Hutchinson Cancer Center, and; 6 Division of Medical Oncology, University of Washington, Seattle, Washington, USA.; 7 Transplantation Research Center, Renal Division, Brigham and Women’s Hospital, Harvard Medical School, Boston, Massachusetts, USA.; 8 Department of Laboratory Medicine and Pathology, University of Minnesota Medical School, Minneapolis, Minnesota, USA.; 9 Division of Hematology/Oncology, Boston Children’s Hospital, Boston, Massachusetts, USA.; 10 Department of Pediatric Oncology, Harvard Medical School, Boston, Massachusetts, USA.; 11 Dana-Farber Cancer Institute, Boston, Massachusetts, USA.; 12 Harvard Medical School, Boston, Massachusetts, USA.

**Keywords:** Immunology, Inflammation, Bone marrow transplantation, Immunotherapy

## Abstract

Chronic graft-versus-host disease (cGVHD) remains a major cause of morbidity and mortality after allogeneic hematopoietic transplantation. cGVHD pathophysiology involves cooperation between T follicular helper cells (TFHs) and germinal center B cells (GCBs), allo- and autoantibody depositions in cGVHD tissues, and fibrosis. We evaluated human CD19–directed chimeric antigen receptor (CAR19) T cell therapy in a clinically relevant murine cGVHD model with bronchiolitis obliterans syndrome (BOS). Although CD8^+^ CAR19 T cells effectively reduced peripheral B cell and GCB frequencies, pulmonary function was unimproved. In contrast, a single infusion of CAR19 CD4^+^ regulatory T cells (Tregs) mitigated ongoing pulmonary disease and modulated germinal centers (GCs) associated with reduced TFH frequencies compared with control Tregs but without measurable B cell depletion. Compared with EGFR Treg infusion, mice receiving CAR19 Tregs exhibited enhanced suppression of B cell activation and preserved splenic architecture, and CAR19 Treg infusion provided greater opportunities for interaction with CD19^+^ B cells at the B cell follicle boundary zones. Taken together with the absence of detectable B cell cytolysis, these findings were most consistent with GC suppression rather than B cell depletion as the dominant mechanism. Overall, our findings suggest that CAR19 Tregs represent a promising and safe cGVHD/BOS therapeutic strategy, offering immunosuppressive benefits and improved disease outcomes that may be more limited with CD8^+^ CAR19 T cell treatment.

## Abstract 2

Human CD19-directed CAR CD4 Tregs suppress chronic Graft-Versus-Host Disease through increased immunosuppressive mechanisms and without overt cytolytic activity against human CD19 B-cells.

## Graphical abstract

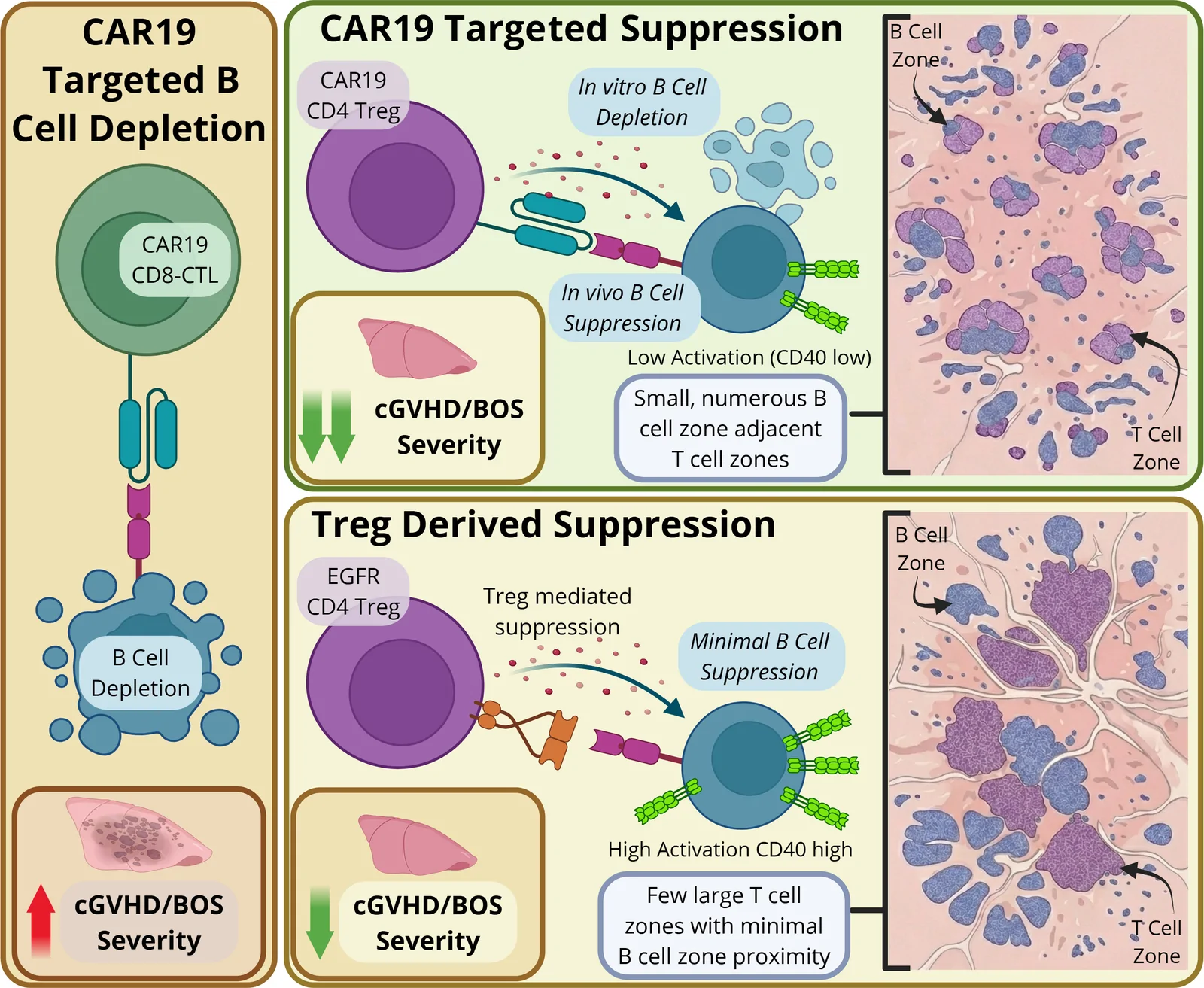

## Introduction

Allogeneic hematopoietic stem cell transplantation (allo-HSCT) remains one of the most effective treatment strategies for hematological disorders and cancers. However, chronic graft-versus-host disease (cGVHD) is a severe complication, affecting 20%–70% of allo-HSCT patients, and remains the leading cause of non-relapse morbidity and mortality (1–4). Among its numerous manifestations, pulmonary cGVHD, or bronchiolitis obliterans syndrome (BOS), often carries the poorest prognosis, with only about 40% of cGVHD/BOS patients surviving 5 years (5, 6).

cGVHD/BOS pathophysiology involves persistent inflammation and dysregulation. Pre-transplant conditioning regimens, donor anti-host alloreactivity, and exuberant T follicular helper cell (TFH) and germinal center B cell (GCB) dysregulation support allo- and autoantibody production that drive target organ tissue fibrosis (7, 8). While B cell depletion strategies such as anti-CD20 mAb (rituximab) show efficacy in some cGVHD settings, their BOS impact is limited, with most studies reporting only stabilization or modest improvement in pulmonary function (9–11). In a murine cGVHD/BOS model, Flynn et al. demonstrated that whereas anti–murine CD20 mAb depleted more than 98% of peripheral B cells, splenic GCB frequencies increased 3-fold and disease remained untreated (8). These findings highlight the need for therapies with access to pathogenic cells and processes in tissues such as the germinal center (GC).

Chimeric antigen receptor (CAR) T cells offer several advantages over mAb therapies. While mAbs are largely restricted to circulation, CAR T cells can infiltrate tissues, including immune-privileged sites. Moreover, mAb efficacy often relies on antibody-dependent cellular cytotoxicity, which may be impaired in immunocompromised patients through delayed natural killer (NK) cell reconstitution or reduced NK activity (12, 13). In contrast, CAR T cells provide their own antigen-specific cytotoxic potential, can mediate long-lasting immune modulation, and may be detectable for more than 10 years (14, 15).

Because allogeneic CD19-directed CAR T cells have treated relapsed or refractory hematological malignancies after HSCT (16–19), we tested CAR19 CD8^+^ T cells to deplete GCBs and suppress cGVHD. While CD8^+^ CAR19 T cells effectively reduced peripheral and GCB frequencies in cGVHD/BOS mice, we observed no improvement in pulmonary function or lung disease burden, suggesting that B cell depletion is insufficient to ameliorate ongoing cGVHD/BOS.

Regulatory T cells (Tregs) help maintain immune homeostasis; their efficacy in preclinical and clinical cGVHD studies is well established (20–24). In murine and human cGVHD, thymus-derived Tregs undergo rapid turnover leading to Treg deficiency (25, 26). In murine cGVHD/BOS, Treg deficiency translates to a paucity of T follicular regulatory cells (TFRs) that otherwise would dampen GC responses (27–31).

CAR19 Tregs mediate context-dependent B cell cytolysis via perforin/granzyme pathways in vitro and in some in vivo models are generally less efficient than CD8^+^ CAR19 T cells (15, 32, 33). Systemic lupus erythematosus studies suggest that the dominant mechanism by which CAR19 Tregs suppress B cells in vivo is non-cytolytic, primarily through TGF-β–dependent and cell contact–dependent suppression (34) including secretion of inhibitory cytokines, metabolic disruption, and modulation of antigen-presenting cell function (35–37). These diverse mechanisms may enable CAR-engineered Tregs to control pathogenic B cell activity in cGVHD/BOS more effectively and with fewer inflammatory consequences such as cytokine release syndrome or immune effector cell–associated neurotoxicity syndrome (ICANS) than conventional cytotoxic T lymphocytes (15, 38).

Here, we assessed the ability of CD8^+^ CAR19 T cells to deplete human CD19–positive (hCD19^+^) transgenic B cells and modulate GC reactions in a murine cGVHD/BOS model. Subsequently we examined whether CAR19 Tregs could improve disease control without CD8^+^ CAR19 T cell side effects. Our results demonstrate a potent effect of CAR19 Tregs in treating established cGVHD/BOS without clinical side effects that can be seen with CAR19 CD8^+^ T cells.

## Results

### CD8^+^ CAR19 T cells failed to treat murine cGVHD/BOS despite peripheral B cell and GCB depletion.

**Figure 1:**
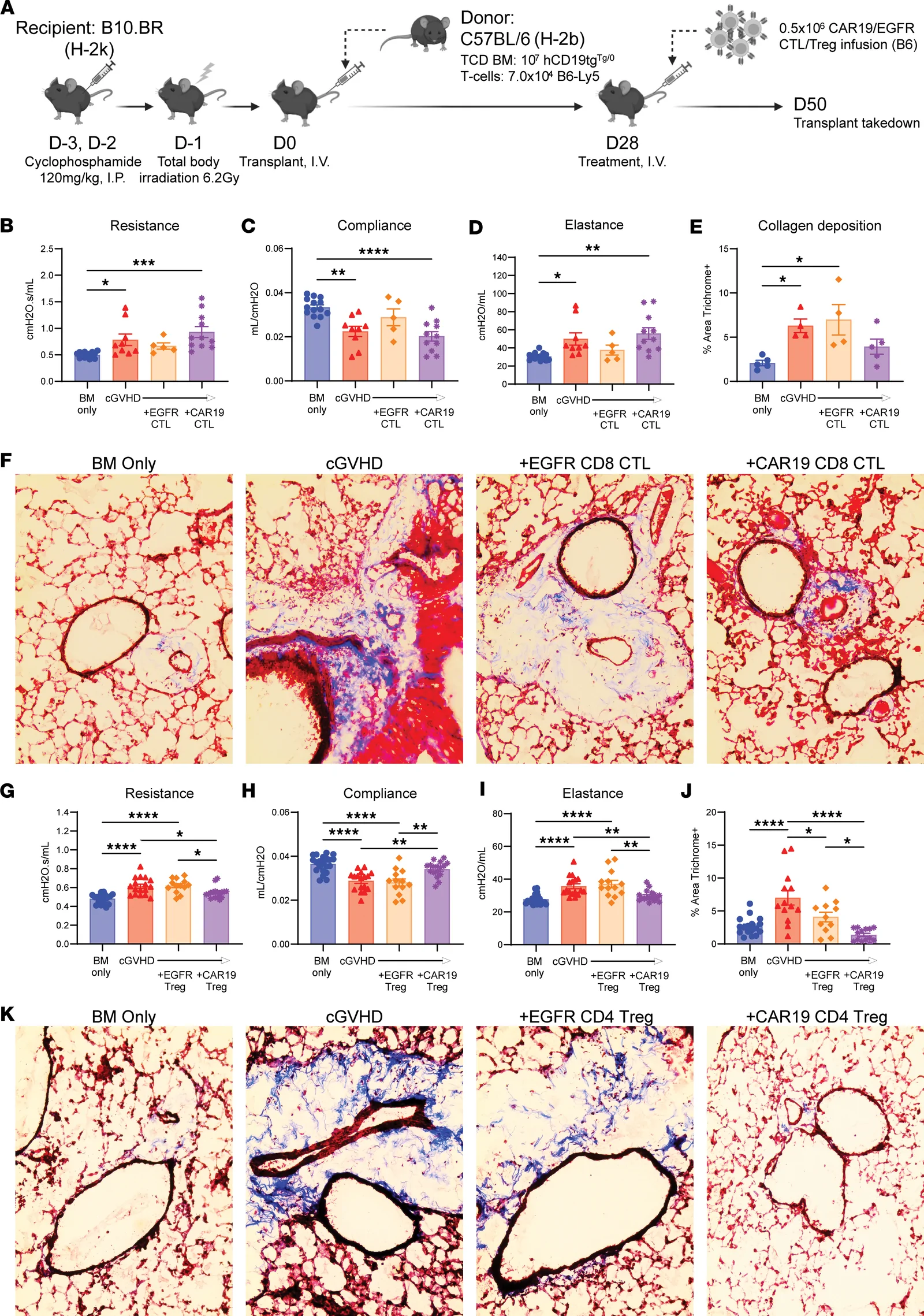
Day 28 CD8^+^ CAR19 T cells do not treat murine cGVHD/BOS, while CAR19 Tregs reduce disease. (**A**) B10.BR mice were conditioned with 120 mg/kg i.p. cyclophosphamide (day –3 and day –2) followed by 6.2 Gy total-body irradiation (day –1). On day 0, recipients received 10^7^ T cell–depleted (TCD) bone marrow (BM) from hCD19tg F1 donors plus 71.5 × 10^4^ purified splenic T cells from C57BL/6 (B6)-Ly5 donors. Groups of cGVHD mice were infused with 0.5 × 10^6^ expanded CD8^+^ CAR19- or EGFR-control effectors on day 28, and on day 50 posttransplant mice were sacrificed for terminal analysis of cGVHD/BOS disease severity. (**B**–**D**) Pulmonary function test data from BM-only (*n* = 14), cGVHD (*n* = 9), EGFR CD8^+^ T cell–treated (*n* = 5), and CAR19 CD8^+^ T cell–treated (*n* = 11) mice including resistance (**B**), compliance (**C**), and elastance (**D**). (**E** and **F**) Quantification of collagen deposition (percentage area trichrome) from Masson’s trichrome–stained lung sections from CD8^+^ CAR19 T cell treatment studies (**E**) with representative images from BM-only (*n* = 5), cGVHD (*n* = 4), EGFR CD8^+^ T cell–treated (*n* = 4), and CAR19 CD8^+^ T cell–treated (*n* = 5) mice (**F**). (**G**–**I**) Pulmonary function test data from BM-only (*n* = 21), cGVHD (*n* = 17), EGFR CD4^+^ Treg–treated (*n* = 13), and CAR19 CD4^+^ Treg–treated (*n* = 17) mice including resistance (**G**), compliance (**H**), and elastance (**I**). (**J** and **K**) Quantification of collagen deposition (percentage area trichrome) from Masson’s trichrome–stained lung sections from CD4^+^ Treg treatment studies (**J**) with representative images from BM-only (*n* = 17), cGVHD (*n* = 11), EGFR CD4^+^ Treg–treated (*n* = 11), and CAR19 CD4^+^ Treg–treated (*n* = 14) mice (**K**).**(F** and **K**) Original magnification, ×200. (**B**–**F**) Data are pooled from 2 independent experiments. (**G**–**K**) Data are pooled from 4 independent experiments. Statistics shown are results of 1-way ANOVA with Tukey’s correction for multiple comparisons. *P < 0.05, **P < 0.01, ***P < 0.001, ****P < 0.0001.

We used a murine multi-organ system cGVHD/BOS model to evaluate CAR19 T cell therapies. B10.BR (H-2k) recipients were conditioned with cyclophosphamide on days –3 and –2 and total-body irradiation on day –1. On day 0, mice received 1 × 10^7^ T cell–depleted (TCD) bone marrow (BM) from C57BL/6 (B6; H-2b) human CD19 (hCD19)–transgenic hemizygous (*hCD19tg^Tg/0^*) donors to rescue hematopoiesis. BM from hemizygous *hCD19tg^Tg/0^* mice was used to reconstitute recipients with *hCD19tg^Tg/0^* BM-derived B cells, enabling testing of donor Tregs and CD8^+^ T cells that express an FDA-approved CAR19 construct consisting of a human CD19 (FMC63) single-chain Fv fragment (scFv), CD8 hinge and transmembrane, human 4-1BB (CD137) costimulatory domain, CD3ζ signaling domain, and functionally inert, truncated EGFR (39). Hemizygous BM donor mice were chosen since hCD19^+^ B cell frequencies are most reflective of human B cell frequencies, whereas homozygous *hCD19tg^Tg/Tg^* mice have 75% fewer circulating B cells (38). A low splenic T cell dose (7 × 10^4^) creates cGVHD/BOS without excessive mortality from chronic alloantigen stimulation (Figure 1A). Flow cytometry confirmed splenic and lung B cell hCD19 expression at day 28 (97.0% and 93.4%, respectively) and day 50 (97.0% and 97.3%, respectively) after transplant (Supplemental Figure 1, A–C; supplemental material available online with this article; https://doi.org/10.1172/jci.insight.196197DS1). cGVHD mice exhibited significantly increased GCB frequencies compared with BM-only controls at both times (Supplemental Figure 1, D–F), validating the model for CAR T cell evaluation.

Splenic CD8^+^ T cells were activated with anti-CD3/CD28 mAb–coated Dynabeads (15, 38) and transduced with hCAR19 or control (truncated EGFR) retrovirus. Transduced CD8^+^ T cells were uniformly enriched to greater than 90% EGFR purity. EGFR and CAR19 expression was confirmed on day 7 before administration (Supplemental Figure 2, A and B). Day 28 infusion of 0.5 × 10^6^ CAR19 or EGFR CD8^+^ T cells did not affect the >80% survival or weights that typically declined by ≤20% from pre–BM transplantation weights, although significant splenic lymphopenia was observed in comparison with cGVHD controls (Supplemental Figure 3, A–C). Day 28 CD8^+^ CAR19 T cells did not improve pulmonary function (Figure 1, B–D) despite significant peripheral B cell and GCB depletion in spleens and lungs (Supplemental Figure 3, D–F).

Spleen immunofluorescent staining on day 50, 22 days after day 28 infusion, showed that CD8^+^ CAR19 T cells decreased overall GC size, with more frequent, fragmented GCs lacking proper CD4^+^ T cell compartmentalization (Supplemental Figure 4, A–C). Splenocyte flow cytometry revealed no TFH, TFR, or TFR/TFH ratio changes (Supplemental Figure 3, G–I). Lung collagen deposition was not significantly reduced (Figure 1, E and F). Despite marked reduction in day 50 lung pathogenic IgG2c deposition (Supplemental Figure 5, A and B), improved clinical outcomes were not seen. Collectively, these data indicate that GCB depletion and reduced IgG2c deposition in the context of donor anti-host reactive allogeneic CD8^+^ CAR19 T cells are insufficient for cGVHD/BOS mitigation.

### CAR19 CD4^+^ Tregs effectively treated murine cGVHD/BOS.

Since CD8^+^ CAR19 T cells did not improve cGVHD/BOS, we explored CAR19 Tregs as an alternative therapy based on previous data demonstrating that CAR19 Tregs had a dual immune suppression and antigen-specific cytolytic capacity without detrimental immune responses inherent to CD8^+^ CAR19 T cells (15, 38). CAR19 Tregs were generated as described previously (15) using FoxP3-GFP reporter mice for flow sorting to 99% purity. Activated Tregs transduced with CAR19- or EGFR-only retrovirus were expanded with Dynabeads in IL-2, maintaining high FoxP3 purity (Supplemental Figure 2, C–E). The same suboptimal dose (0.5 × 10^6^) used for CD8^+^ CAR19 T cells was infused into day 28 cGVHD mice to intentionally minimize known beneficial effects with high-dose Tregs and facilitate detection of differences between groups (20, 21, 40).

Neither EGFR nor CAR19 Tregs adversely affected overall survival or weights (Supplemental Figure 6, A and B). In contrast to CD8^+^ EGFR or CAR19 T cells, CAR19 Tregs significantly improved pulmonary function across resistance, compliance, and elastance parameters (Figure 1, G–I) and significantly reduced lung collagen deposition in comparison with cGVHD controls and EGFR Treg–treated mice (Figure 1, J and K). CAR19 Treg–treated mice exhibited reduced pathology in the colon and liver, organs shown to be cGVHD target organs in this model (Supplemental Figure 7, A and B). However, lung histopathology scores were not reduced with either treatment. Possible explanations include the fact that histopathology scoring examines only a small fraction of lung tissue, whereas lung function measures the respiratory capacity of the lung as an entire organ, and lung injury repair, as assessed by histopathological scores, may require a longer time period than lung function to be readily detectable. These results demonstrate that CAR19 Tregs significantly alleviated murine cGVHD/BOS with multi-organ system disease.

### CAR19 Treg–mediated treatment of cGVHD/BOS did not require GCB depletion.

We sought to determine the mechanism(s) by which CAR19 Tregs improved cGVHD/BOS. Previously we reported that non-transplanted, day –1 cyclophosphamide-conditioned *hCD19tg^Tg/0^* mice experienced a profound and equivalent hCD19^+^ B cell depletion on day 5 after CAR19 Treg or CD8^+^ CAR19 T cell infusion; each had a potent effect on reducing B cell lymphoma (38). In an acute GVHD model, lethally irradiated *hCD19tg^Tg/0^* recipients of BALB/c (H-2d) donor BM with or without purified T cells with or without CD8^+^ EGFR or CAR19 T cells or EGFR Tregs died of GVHD with survival rates of 0%–20%, in contrast to 60%–100% survival in CAR19 Treg–treated recipients, most consistent with CAR19 Treg–mediated donor T cell alloresponse suppression (15). These data reflect CAR Treg cytolytic and suppressive properties, offering 2 possible mechanisms (B cell depletion and immunosuppression) for CAR19 Treg efficacy in cGVHD/BOS mice.

**Figure 2:**
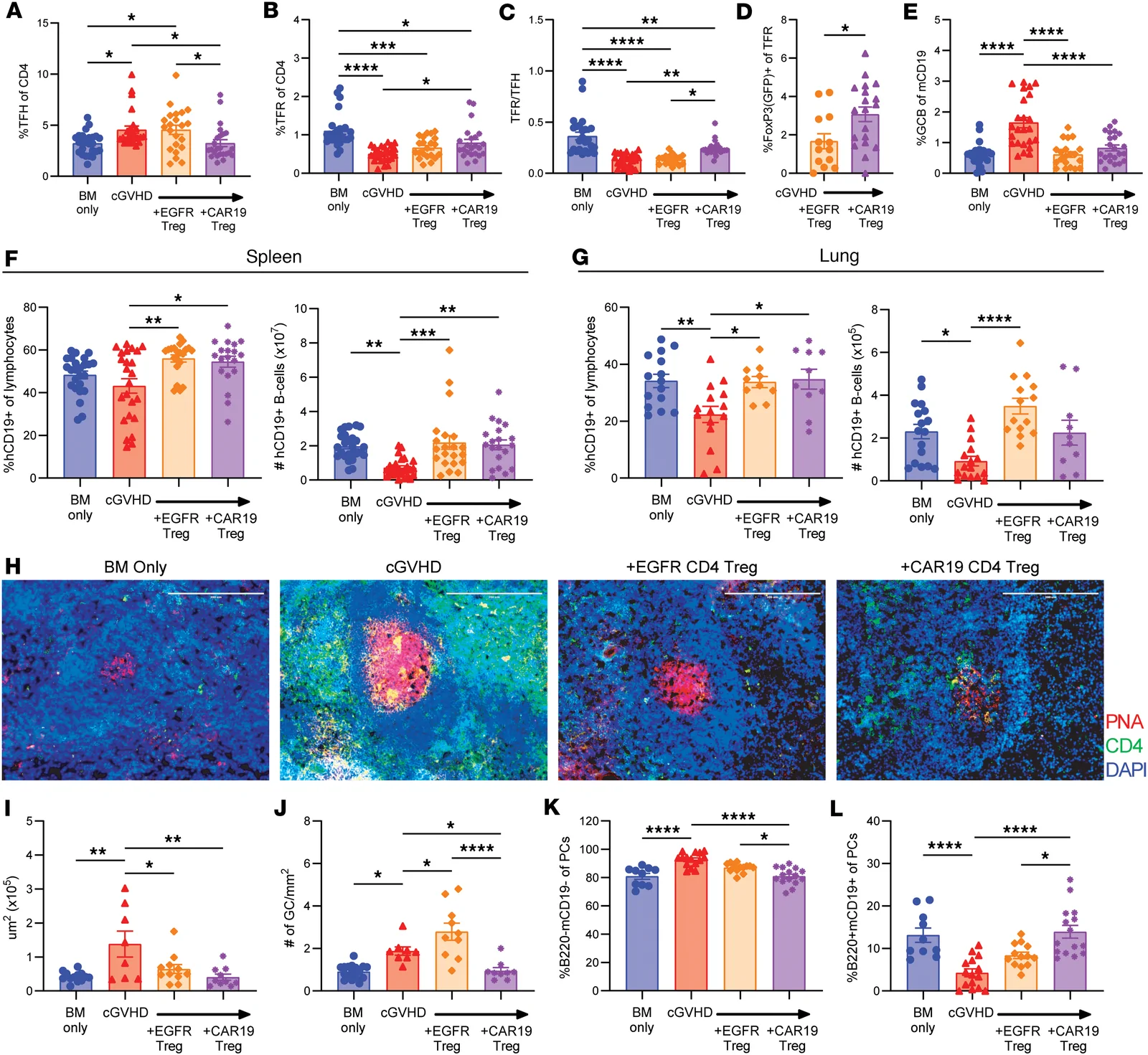
CAR19 Tregs inhibit GC reactions in murine cGVHD/BOS. (**A**–**E**) Day 50 spleens from cGVHD mice treated with EGFR or CAR19 Tregs were analyzed for frequency of TFHs (FoxP3^–^CXCR5^+^PD-1^+^BCL6^+^) of CD4^+^ (**A**), frequency of TFRs (FoxP3^+^CXCR5^+^PD-1^+^BCL6^+^) of CD4^+^ (**B**), TFH/TFR ratio (**C**), frequency of FoxP3(GFP)^+^ infused Tregs of TFRs (**D**), and frequency of GCBs of mouse CD19 positive (mCD19^+^) (**E**). (**F** and **G**) Frequency and counts of hCD19^+^ B cells of lymphocytes in day 50 spleen (**F**) and lung (**G**). (**H**) Representative images (original magnification, ×200) of spleen sections stained for GCs with peanut agglutinin (PNA; red), CD4 (green), and DAPI (blue). Scale bars: 200 μm. (**I** and **J**) Quantification of GC sizes (**I**) and frequencies per mm^2^ (**J**). (**K** and **L**) Day 50 spleens were analyzed for frequencies of mature B220^–^mCD19^–^ plasma cells (PCs) of total CD138^+^BLIMP1^+^ PCs (**K**) or immature B220^+^mCD19^+^ PCs of total PCs (**L**). Data are pooled from 2–4 independent experiments. (**A**–**C**, **E**, and **F**) Day 50 spleens were analyzed from BM-only (*n* = 26), cGVHD (*n* = 25), EGFR CD4^+^ Treg–treated (*n* = 21), and CAR19 CD4^+^ Treg–treated (*n* = 23) mice. (**D**) Day 50 spleens were analyzed from EGFR CD4^+^ Treg–treated (*n* = 17) and CAR19 CD4^+^ Treg–treated (*n* = 25) mice. (**G**) Day 50 lungs were analyzed from BM-only (*n* = 17), cGVHD (*n* = 16), EGFR CD4^+^ Treg–treated (*n* = 14), and CAR19 CD4^+^ Treg–treated (*n* = 10) mice. (**I** and **J**) Day 50 spleens were analyzed from BM-only (*n* = 13), cGVHD (*n* = 8), EGFR CD4^+^ Treg–treated (*n* = 11), and CAR19 CD4^+^ Treg–treated (*n* = 10) mice. (**K** and **L**) Day 50 spleens were analyzed from BM-only (*n* = 10), cGVHD (*n* = 15), EGFR CD4^+^ Treg–treated (*n* = 13), and CAR19 CD4^+^ Treg–treated (*n* = 15) mice. Statistics shown are results of 1-way ANOVA with Tukey’s correction for multiple comparisons. *P < 0.05, **P < 0.01, ***P < 0.001, ****P < 0.0001.

In cGVHD/BOS mice, CAR19 Treg infusion reduced TFH frequencies, increased TFR frequencies, and elevated TFR/TFH ratios in comparison with cGVHD controls (Figure 2, A–C). In contrast, EGFR Treg–treated cGVHD mice had TFH frequencies and TFR/TFH ratios similar to those of cGVHD controls. TFR frequencies were comparable to those of CAR19 Treg–treated mice, possibly reflecting Treg infusion. Although EGFR and CAR19 Treg spleen and lung frequencies and numbers were similar (Supplemental Figure 8, A–C), CAR19 Tregs increased TFRs by inhibiting cGVHD associated with reduced TFR frequencies or directly contributing GC TFRs (Figure 2D). These data are consistent with observations above that CAR19 Treg infusion augmented TFR/TFH ratios relative to EGFR Tregs (Figure 2C).

Contrary to our expectation that CAR19 Tregs would more efficiently deplete GCBs compared with EGFR Tregs and be superior in treating cGVHD/BOS, CAR19 and EGFR Tregs similarly reduced GCB frequencies relative to cGVHD/BOS controls (Figure 2E). CAR19 and EGFR Tregs increased total splenic hCD19^+^ B cell frequencies and numbers to levels comparable to those in BM-only controls, consistent with Treg-mediated mitigation of cGVHD-induced B cell lymphopenia (Figure 2, F–H). However, CAR19 Tregs reduced the overall cGVHD-induced increase in GC size and frequency in comparison with cGVHD controls and EGFR Tregs (Figure 2, I and J).

Day 50 splenic plasma cell (PC) frequencies were analyzed as a possible source of high pathogenic antibody levels that might be differentially susceptible to CAR Treg–mediated suppression (Figure 2, K and L). CAR19 Tregs decreased mature PC frequencies and increased immature PC frequencies in comparison with cGVHD controls and EGFR Tregs, approaching BM-only control levels. Reduction of mature PCs was associated with significantly decreased lung pathogenic IgG2c deposition (Supplemental Figure 9, A and B).

Since we could not exclude rapid *hCD19tg^Tg/0^* B cell depletion by CAR19 Tregs that may have been missed on day 50, cGVHD/BOS recipients were studied on day 35, 7 days after EGFR or CAR19 Treg infusion, for evidence of *hCD19tg^Tg/0^* B cell depletion seen in cyclophosphamide-treated mice (38). Unexpectedly, no measurable B cell depletion was observed with CAR or EGFR Tregs in spleen or lungs (data not shown). Specifically, there were no frequency or count differences between CAR19 and EGFR Tregs in these tissues early post-infusion (data not shown). Together, these findings suggest that CAR19 Tregs suppress pathological GC reactions without depleting B cells, resulting in reduced cGVHD/BOS, enhanced B cell recovery, and prevention of secondary lymphoid organ involution typically seen in untreated cGVHD recipients.

### CAR19 Tregs maintained cytolytic capacity in vitro and in cyclophosphamide-treated syngeneic recipients.

**Figure 3:**
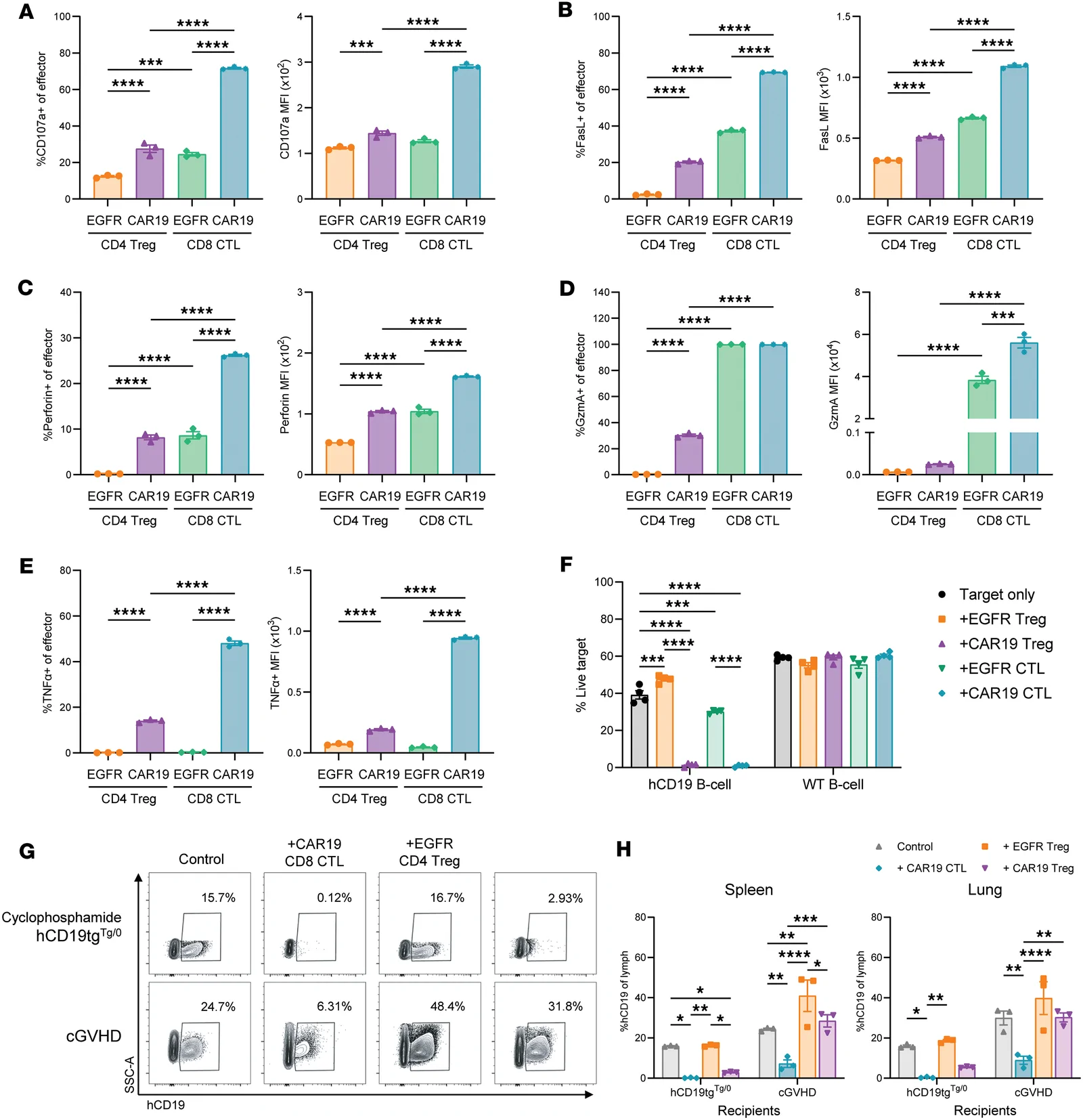
CAR19 Tregs maintain cytolytic capacity in vitro and in cyclophosphamide-treated syngeneic recipients. (**A**–**E**) Frequency and MFI of cytolytic markers after EGFR CD4^+^ Treg (*n* = 3), CAR19 CD4^+^ Treg (*n* = 3), EGFR CD8^+^ cytotoxic T lymphocyte (CTL) (*n* = 3), and CAR19 CD8^+^ CTL (*n* = 3) coculture with freshly isolated hCD19^+^ B cells at 1:5 effector/B cell ratio for 5–48 hours: (**A**) CD107a (5 hours), (**B**) FasL (48 hours), (**C**) perforin (24 hours), (**D**) granzyme A (48 hours), (**E**) TNF-α (24 hours). (**F**) Forty-eight-hour in vitro flow killing assay of freshly isolated *hCD19tg^Tg/0^* or wild-type (WT) B cells cocultured with EGFR CD4^+^ Tregs (*n* = 4), CAR19 CD4^+^ Tregs (*n* = 4), EGFR CD8^+^ CTLs (*n* = 4), and CAR19 CD8^+^ CTLs (*n* = 4) at a ratio of 5 effectors to 1 target with addition of 5 μg/mL αIgM and 100 ng/mL BAFF. (**G** and **H**) cGVHD/BOS mice were set up as previously described and infused with 3.0 × 10^6^ effectors on day 28 (Figure 1A). Syngeneic *hCD19tg^Tg/0^* mice were treated with 300 mg/kg cyclophosphamide the day before infusion of 3.0 × 10^6^ effectors. Spleen and lung tissues were harvested 5 days after infusion for flow analysis. Representative flow cytometry plots of hCD19^+^ B cells of lymphocytes from spleen tissues (**G**) with bar graph summary of spleen (left) and lung (right) tissues (**H**) from in vivo killing experiment in control (no treatment) (*n* = 3), CAR19 CD8^+^ CTL–treated (*n* = 3), EGFR CD4^+^ Treg–treated (*n* = 3), and CAR19 CD4^+^ Treg–treated (*n* = 3) mice. Data are representative of 2–3 independent experiments. Statistics shown are results of 1-way ANOVA with Tukey’s correction for multiple comparisons. *P < 0.05, **P < 0.01, ***P < 0.001, ****P < 0.0001.

To better understand why CAR19 Tregs did not deplete B cells in cGVHD/BOS, we reevaluated CAR19 Treg cytolytic potential against B cells. We cocultured EGFR or CAR19 Tregs and CD8^+^ T cells for 5–48 hours with *hCD19tg^Tg/0^* B cells (Figure 3, A–E, and Supplemental Figures 10–12). CAR19 Tregs cocultured with and activated by *hCD19tg^Tg/0^* B cells exhibited higher baseline CD107a and perforin expression compared with EGFR Tregs, and upregulated CD107a, FasL, perforin, granzyme A (but not granzyme B), TNF-α, and IFN-γ upon CAR stimulation, albeit at lower levels than CD8^+^ CAR19 T cells. These results confirm that CAR19 Tregs possess an antigen-specific cytolytic capacity with a distinct effector profile.

To quantify CAR19 Treg–mediated B cell cytolysis, we performed a flow cytometry–based killing assay by coculturing naive hCD19^+^ or wild-type B cells with EGFR or CAR19 Tregs at a 5:1 ratio of Tregs to B cells for 48 hours. Controls were CD8^+^ EGFR or CAR19 CD8^+^ T cells. B cells were activated with anti-IgM and B cell–activating factor (BAFF). CAR19 Tregs and CD8^+^ CAR19 T cells demonstrated robust killing of hCD19^+^ B cells, with an average of 1.3% and 0.85% live B cells remaining, respectively, compared with 39.1% in control cultures without effectors (Figure 3F). CAR19 Treg killing was hCD19 antigen specific with no cytolysis observed against wild-type B cells; EGFR Tregs showed no cytolysis under any condition.

To assess in vivo cytolytic capacity, *hCD19tg^Tg/0^* recipients were pretreated with cyclophosphamide, infused with 3 × 10^6^ syngeneic CAR19 Tregs or CD8^+^ CAR19 T cells (38), and set up in parallel with the cGVHD/BOS model. This ensured that day 28 cGVHD CAR19 Treg or CD8^+^ CAR19 T cell infusion coincided with day 0 infusion of the same cell aliquot given to non-transplanted, cyclophosphamide-treated mice (38). By day 5, splenic hCD19^+^ B cell frequencies decreased from 16.0% in non-infused mice to 0.20% and 2.93% in cyclophosphamide-treated mice receiving CD8^+^ CAR19 T cells and CAR19 Tregs, respectively, with similar lung B cell aplasia (Figure 3, G and H). In contrast, in cGVHD/BOS mice, only CD8^+^ CAR19 T cells reduced splenic hCD19^+^ B cell frequencies (from 24.3% to 7.2%), whereas CAR19 Tregs had no effect. These results demonstrate that the same CAR19 Treg aliquot retained B cell elimination capacity in vitro and in cyclophosphamide-treated, non–BM transplantation recipients but not in cGVHD/BOS mice.

### CAR19 Treg cytolysis was uninhibited by cGVHD serum and required cell-cell contact.

**Figure 4:**
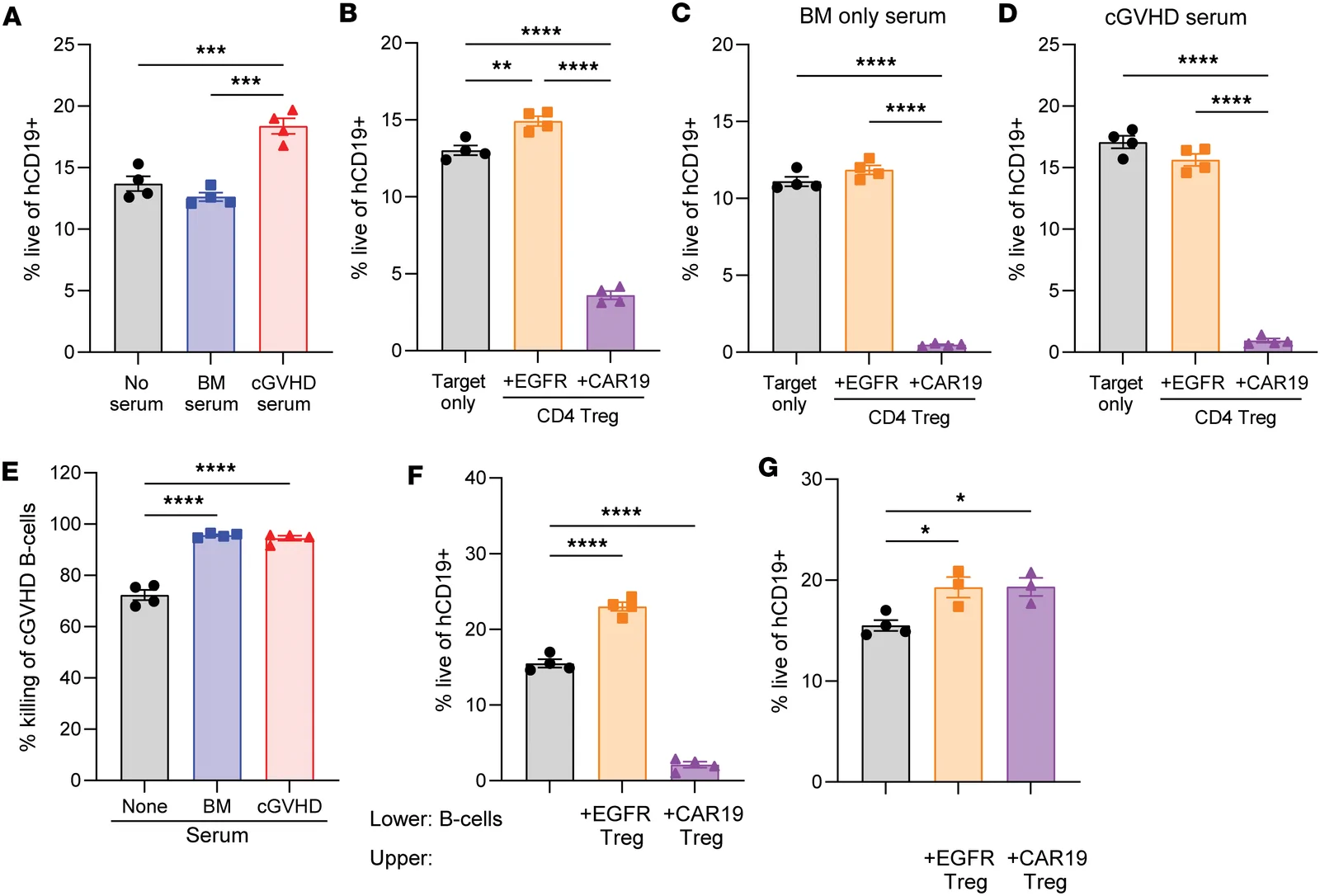
CAR19 Treg cytolytic activity is uninhibited by cGVHD/BOS serum and requires cell-cell contact. (**A**–**G**) All in vitro killing assays were cultured for 48 hours with ex vivo day 28 cGVHD/BOS B cells at an effector/target ratio of 5:1. (**A**) Frequency of live cGVHD B cells in target-only cultures of the killing assay following 1:10 addition of BM-only or cGVHD serum. (**B**–**D**) In vitro flow killing assay of cGVHD B cells by CAR19 versus EGFR Tregs with no serum (**B**) or 1:10 serum addition from BM-only mice (**C**) or cGVHD/BOS mice (**D**). (**E**) Frequency of CAR19 Treg killing of cGVHD B cells from **B**–**D**. (**F** and **G**) In vitro flow killing assay in a Transwell plate with freshly isolated *hCD19tg^Tg/0^* B cells and addition of 5 μg/mL αIgM and 100 ng/mL BAFF. hCD19^+^ B cells were added to lower wells. Effectors were added to the lower wells (**F**) or upper inserts (**G**). Data are representative of 3 independent experiments. (**A**–**F**) Groups were analyzed from B cell–only (*n* = 4), EGFR CD4^+^ Treg (*n* = 4), and CAR19 CD4^+^ Treg (*n* = 4) cocultures. (**G**) Groups were analyzed from B cell–only (*n* = 4), EGFR CD4^+^ Treg (*n* = 3), and CAR19 CD4^+^ Treg (*n* = 3) cocultures. Statistics shown are results of 1-way ANOVA with Tukey’s correction for multiple comparisons. *P < 0.05, **P < 0.01, ***P < 0.001, ****P < 0.0001.

Next, in order to determine whether GCBs from cGVHD/BOS were resistant to CAR19 Treg cytolysis, or whether soluble factors in the in vivo cGVHD environment might account for the lack of profound CAR19 Treg–mediated B cell killing, a 48-hour flow cytometry–based killing assay was used. B cells isolated from day 28 cGVHD/BOS mice were cocultured at a 5:1 Treg/B cell ratio, with or without serum from day 28 BM-only or cGVHD /BOS mice (1:10 dilution). Adding cGVHD serum significantly increased baseline survival of ex vivo cGVHD B cells compared with no serum or BM-only serum (Figure 4A). CAR19 Tregs still mediated significant killing of ex vivo cGVHD B cells, with an average of 3.6% live B cells remaining compared with 13.0% in control cultures without effectors (Figure 4B). EGFR Tregs showed no cytolytic activity. Importantly, CAR19 Tregs maintained robust cytolysis in the presence of BM-only and cGVHD serum (Figure 4, C and D), with no significant difference between serum groups (Figure 4E).

We investigated whether in vitro CAR19 Treg cytolysis required direct cell-cell contact. Using Transwell plates, *hCD19tg^Tg/0^* naive B cells were plated in the lower chambers with effectors in the same chamber or separated by a membrane at 5:1 effector/B cell ratios. Robust cytolysis was observed only when CAR19 Tregs or CD8^+^ CAR19 T cells were in direct contact with B cells; no killing occurred when separated, and B cell survival increased (Figure 4, F and G), indicating that CAR19 Treg cytolysis is contact dependent. Together, these findings indicate that CAR19 Tregs are fully cytolytic via contact-dependent mechanisms, and neither cGVHD B cell resistance nor soluble factors in cGVHD/BOS serum inhibited CAR19 Treg cytolysis ex vivo.

### CAR19 Tregs directly interacted with B cells and inhibited B cell activation in cGVHD/BOS.

**Figure 5:**
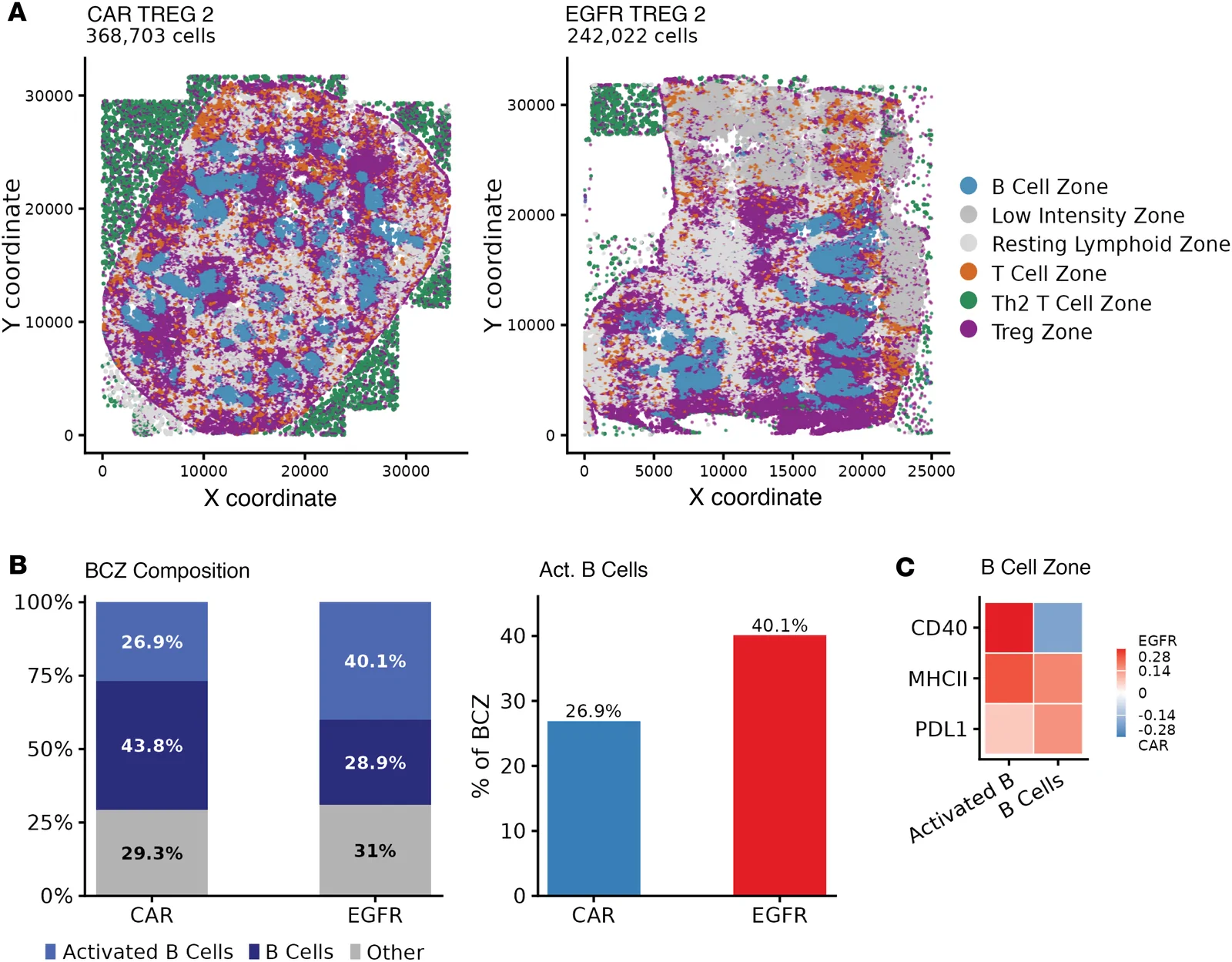
Day 28 CAR19 Tregs predominantly localize to follicular T cell–rich areas instead of B cell–rich areas in cGVHD/BOS spleens. (**A**) Annotated spatial domains detected using local indicators of spatial association (LISA) functions for each cell at multiple radii followed by manual annotation based on cell type distribution patterns. (**B**) Proportions of B cells versus activated B cells in B cell follicle spatial domain. Left: Frequencies of total cells in domains. Right: Direct comparison of activated B cell frequency. BCZ, B cell zone. (**C**) Heatmap of B cell–associated marker mean intensity differences between CAR19 and EGFR Treg–treated mice. Color maps to fold change between conditions. Increased fold change (red) was seen for most markers in EGFR samples. The left column is fold change difference by marker in activated B cells, while the right column is for B cells. Data analysis was performed on a single slide for each condition.

To better understand the mechanism(s) by which CAR19 Tregs treat cGVHD/BOS, we used spatial proteomics to deconvolute cell-cell interactions in cGVHD/BOS (Supplemental Figure 13A). On day 33, 5 days after Treg infusion into cGVHD/BOS mice, spatial proteomics identified distinct Treg localization patterns between mice given EGFR and CAR19 Tregs. Compared with mice given EGFR Tregs, spleens from mice given CAR19 Tregs demonstrated a higher infused Treg proportion surrounding B cell follicles, based on individual cell type deconvolution and annotation (Supplemental Figure 13, B and C) and spatial domain analysis (Figure 5A). B cell subtyping within B cell zones revealed an approximately 1.5-fold decrease in the proportion of CD40^+^ (activated) B cells in CAR19 compared with EGFR Treg–treated mice, with a reciprocal approximately 1.5-fold increase in naive (non-activated) B cells (Figure 5B). Of CD40^+^ B cells, the mean intensity of CD40 expression was about 25% greater in EGFR Treg mice compared with CAR19 Treg mice (Figure 5C).

**Figure 6:**
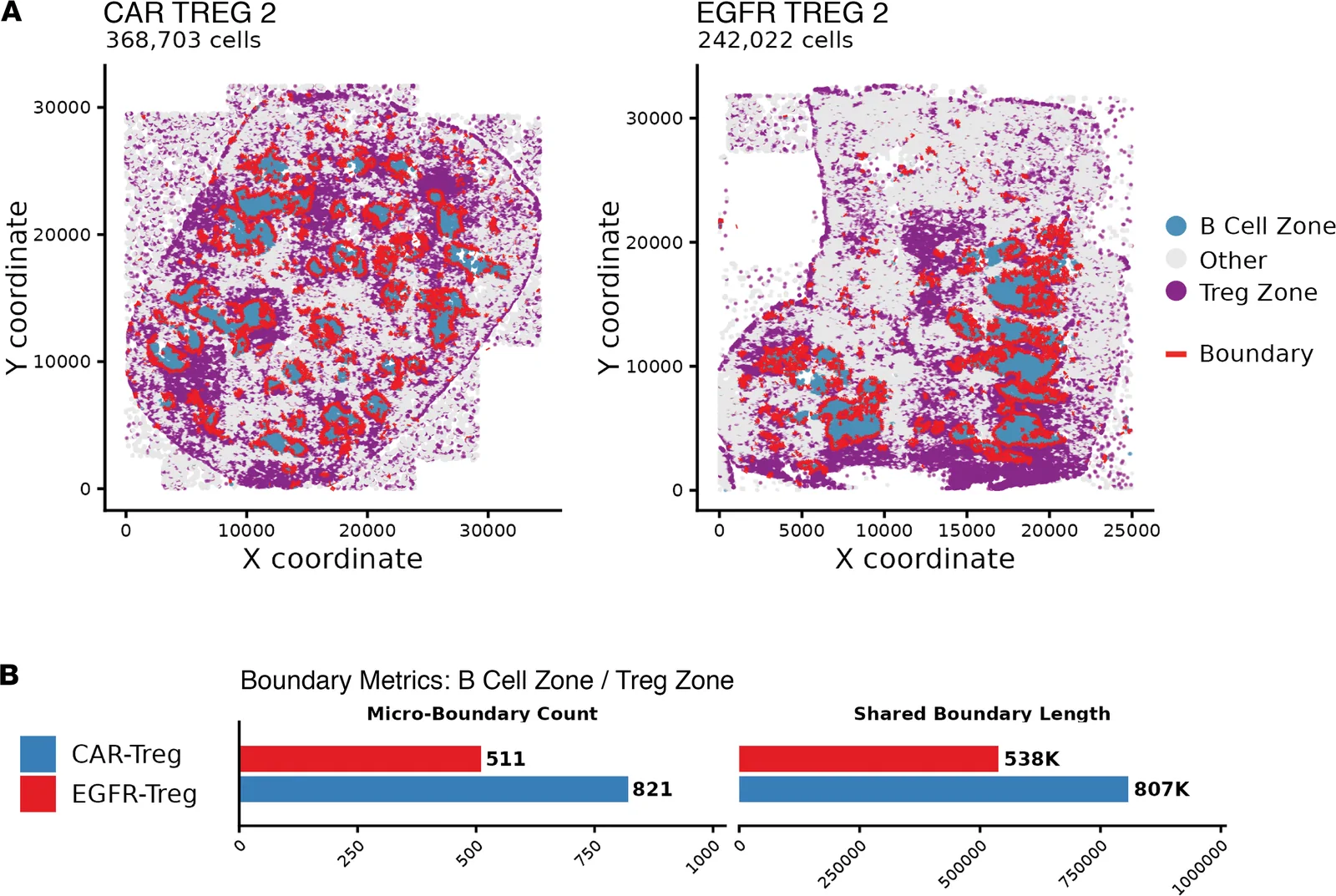
CAR19 Treg–enriched zones show greater interaction boundaries with follicular B cell zones than EGFR Treg controls. (**A**) Secondary annotation of B cell zone and Treg zone spatial domains from Figure 5 with inclusion of detected boundaries of interaction between them. (**B**) Quantifications of B cell and Treg zone boundaries of interaction. Left: Number of individual boundaries detected. Right: Total combined boundary length in pixels. Data analysis was performed on a single slide for each condition.

Visualization of direct CAR19 Treg and B cell colocalization was not observed, possibly reflecting earlier or transient effects. Therefore, we addressed larger spatial interactivity patterns by evaluating splenic B cell follicle and Treg-enriched spatial zone boundary regions (Figure 6A), serving as a proxy for interaction opportunities between these cell types. Compared with EGFR Treg mice, CAR19 Treg mice had approximately 2-fold as many boundaries with an approximately 1.5-fold greater shared interaction length between B cell follicles and Treg-enriched zones (Figure 6B). These spatial proteomics results demonstrate that CAR19 Treg localization is distinct from EGFR Tregs, and that the splenic architecture in CAR19 Treg–treated mice would provide more opportunities for CD19^+^ B cell interaction at the B cell follicle boundary zones. These interactions may drive Treg-mediated suppression of B cell activation.

### CAR19 Tregs exhibited a more robust suppressive profile compared with EGFR Tregs.

**Figure 7:**
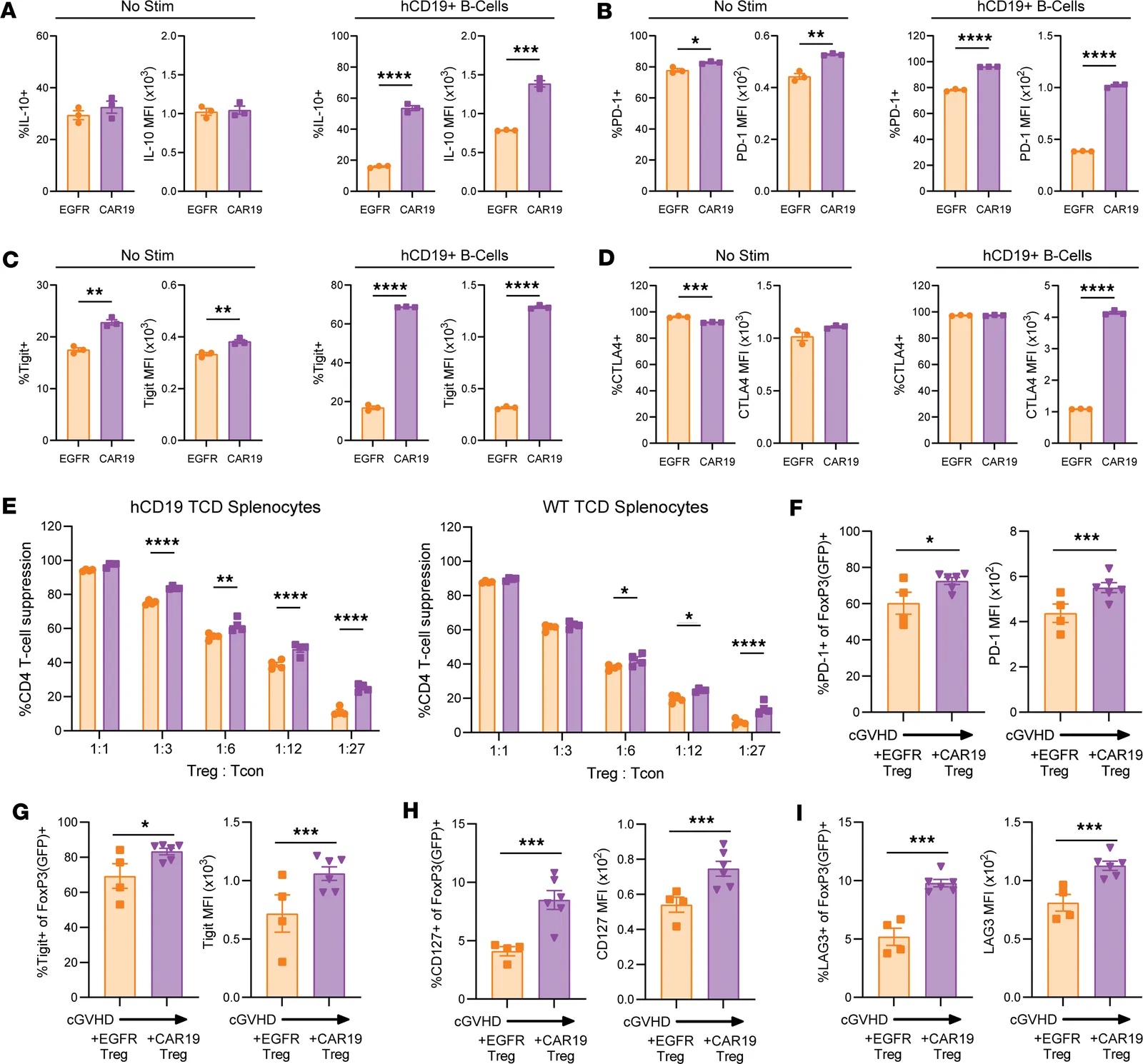
CAR19 Tregs have greater suppressive capacity compared with EGFR Tregs in vitro and in vivo during murine cGVHD/BOS. (**A**–**D**) Frequency and MFI of suppressive markers expressed by EGFR CD4^+^ Tregs (*n* = 3) or CAR19 CD4^+^ Tregs (*n* = 3) after 48 hours of no stimulation or coculture with *hCD19tg^Tg/0^* B cells: (**A**) IL-10, (**B**) PD-1, (**C**) Tigit, (**D**) CTLA-4. (**E**) In vitro suppression of CD4^+^ T cell proliferation by EGFR Tregs (*n* = 4) or CAR19 Tregs (*n* = 4) cocultured with hCD19^+^ TCD splenocytes (left) or wild-type TCD splenocytes (right). (**F**–**I**) Frequency and MFI of infused FoxP3(GFP)^+^ Tregs from day 50 spleens of cGVHD mice treated with EGFR Tregs (*n* = 4) or CAR19 Tregs (*n* = 6) expressing PD-1 (**F**), Tigit (**G**), CD127 (**H**), and LAG3 (**I**). Data are representative of 3 independent experiments. Statistics shown are results of 1-way ANOVA with Tukey’s correction (**A**–**D** and **F**–**I**) or 2-way ANOVA with Šidák’s correction for multiple comparisons (**E**). *P < 0.05, **P < 0.01, ***P < 0.001, ****P < 0.0001.

With perceived CAR19 Treg–B cell interaction increases, we investigated whether such interactions may render CAR19 Tregs more suppressive after CD19 engagement. We cocultured CAR19 or EGFR Tregs with *hCD19tg^Tg/0^* naive B cells at a 1:5 Treg/B cell ratio for 5–48 hours (Figure 7, A–D, and Supplemental Figure 14). Notably, CAR19 Tregs exhibited higher baseline PD-1 and TIGIT expression compared with EGFR Tregs. Upon CAR stimulation by hCD19^+^ B cells, CAR19 Tregs showed increased frequencies and mean fluorescence intensities (MFIs) for molecules associated with suppression, including IL-10, PD-1, and TIGIT, and elevated CTLA-4 MFI relative to EGFR Tregs.

In vitro suppression assays were performed to compare CAR19 and EGFR Treg potency. Treg groups were cocultured at varying ratios with congenic conventional T cells (Tcons) labeled with CellTrace CFSE proliferation dye. TCD splenocytes from *hCD19tg^Tg/0^* or wild-type B6 mice were added at 1:1 with Tcons. CAR19 Tregs demonstrated superior suppression of T cell proliferation compared with EGFR Tregs, with more consistent and greater suppression when cultured with hCD19^+^ TCD splenocytes (Figure 7E).

Flow cytometry analysis of day 50 cGVHD/BOS spleens revealed that day 28 CAR19 Treg infusion exhibited a more suppressive and memory-like phenotype compared with EGFR Tregs, indicated by increased frequencies and MFIs of TIGIT, PD-1, LAG3, CD127, and KLRG1 (Figure 7, F–I, and Supplemental Figure 15).

Collectively, these findings highlight that CAR19 Tregs are more suppressive than EGFR Tregs in vitro and in vivo, and that their suppressive phenotypes can be effectively mediated through CAR signaling.

## Discussion

We report that CAR19 CD8^+^ T cells effectively depleted peripheral and GC B cells but were unable to alleviate pulmonary disease in cGVHD/BOS mice. In contrast, a single CAR19 CD4^+^ Treg infusion significantly improved pulmonary disease. CAR19 Tregs suppressed GC reactions without measurable depletion of peripheral or GC B cells, instead exhibiting enhanced suppressive capacity and preferential B cell–rich follicular zone localization rather than T cell–rich areas. Altogether, our findings suggest that CAR19 Tregs ameliorate cGVHD/BOS primarily through immunosuppression rather than cytolysis.

We proposed that CD8^+^ CAR19 T cells could achieve effective tissue infiltration and deplete GCBs to treat cGVHD/BOS to a greater extent than observed with anti-CD20 mAb (rituximab). Most studies reported only stabilization or modest improvement in pulmonary function (9–11), and our own studies showed that despite anti-CD20 mAb–induced depletion of more than 98% of peripheral B cells, B cells remained in the lung and cGVHD/BOS was unmitigated (8). CD8^+^ CAR19 T cells effectively depleted peripheral and tissue-localized GCBs, resulting in decreased IgG2c deposition in cGVHD/BOS lungs that we expected would improve disease burden. Instead, CD8^+^ CAR19 T cell targeting of splenic B cells was accompanied by fragmented and incomplete GC formation, consistent with disruption and abolishment in human lymph node follicular structures in CAR19 T cell–treated autoimmune patients (41). This architectural disruption likely impaired high-affinity, class-switched antibodies including pathogenic IgG2c in this model. Nonetheless, CD8^+^ CAR19 T cell treatment did not improve disease in cGVHD/BOS mice, as evidenced by lung dysfunction, persistent tissue inflammation, fibrosis, and ongoing immune dysregulation.

Expanded murine CAR19 Tregs were cytolytic as demonstrated via in vitro assays and in vivo assays. CAR19 Tregs depleted peripheral B cells in cyclophosphamide-treated, non-transplanted, and acute GVHD mice (15, 38), each of which had *hCD19tg^Tg/0^* B cells, leading to the possibility that CAR19 Tregs could directly deplete peripheral and GC B cells. Here, we confirmed that CAR19 Tregs acquired a cytolytic profile upon antigen-specific activation, though at lower levels than CD8^+^ CAR19 T cells, consistent with other reports (32). Moreover, we show that CAR19 Treg aliquots from the same culture simultaneously given to cGVHD/BOS mice reconstituted with donor BM-derived *hCD19tg^Tg/0^* B cells and cyclophosphamide-conditioned *hCD19tg^Tg/0^* mice resulted in profound B cell depletion within 5 days after infusion, the time chosen for B cell depletion analysis in cGVHD/BOS mice. In preliminary studies, the lack of B cell depletion did not appear to be due to inadequate CAR19 Treg dosing, since even higher CAR19 Treg doses failed to deplete cGVHD/BOS B cells.

We also found that although CD8^+^ CAR19 T cells were able to effectively deplete both peripheral and GC B cells, these depletions were accompanied by fragmented and incomplete GC formations, consistent with clinical histopathology showing that CAR19 T cells, but not anti-CD20 mAb treatment of autoimmune disorder patients, disrupts and abolishes human lymph node follicular structures (41). Since the extrafollicular and GC locations are the primary sites for B cell class switching and affinity maturation occurs, this architectural disruption likely impairs high-affinity, class-switched antibody generation. Accordingly, we observed a decrease in pathogenic IgG-switched IgG2c deposited in cGVHD/BOS lungs following CD8^+^ CAR19 T cells, which would be expected to improve disease burden. However, this was not the case, a finding that may be attributable to persistent tissue inflammation, ongoing immune dysregulation, and underlying fibrosis.

The lack of B cell depletion by CAR19 Tregs given to cGVHD/BOS mice was not due to impaired migration into spleen or lungs, since CAR19 and EGFR Treg frequencies and numbers were similar when analyzed at 7 days or 22 days after infusion. Ex vivo CAR19 Tregs efficiently eliminated cGVHD B cells, proving that these B cells were not intrinsically resistant to CAR19 Treg–mediated cytolysis nor was killing inhibited by addition of cGVHD serum to the killing assay. This context-dependent effect is consistent with recent studies showing that CAR19 Tregs can mediate B cell depletion in some settings but primarily suppress B cell function via non-cytolytic mechanisms in chronic disease models (32, 34). Such findings underscore the potential added benefits of therapeutic approaches that also restore immune regulation and tolerance. Supportive evidence provided here shows that CAR19 Tregs improved pulmonary function and reduced fibrosis, lung IgG2c deposition, PC differentiation, and TFH frequencies, thereby increasing the TFR/TFH ratio.

Several studies demonstrate that CAR Treg–mediated suppression typically predominates over cytolytic activity, particularly in established autoimmune and inflammatory settings (32, 34, 42, 43). Imura et al. demonstrated in a xenograft GVHD model that Treg suppression of B cell responses was primarily mediated by TGF-β–dependent pathways and modulation of TFH and TFR ratios, rather than direct B cell killing (34). In a systemic lupus erythematosus model, CAR19 Tregs overexpressing FoxP3 suppressed B cell proliferation and autoantibody production in an antigen-specific manner, without detectable cytotoxicity, acting primarily through immunomodulation at B cell activity sites (42). These CAR19 Tregs stabilized circulating B cell frequencies and delayed B cell lymphopenia, thereby reshaping the B cell compartment. We observed similar CAR19 Treg treatment results in cGVHD/BOS, wherein CAR19 Treg therapy increased overall B cell frequencies compared with the B cell lymphopenia seen in cGVHD/BOS mice that were untreated or treated with CD8^+^ CAR19 T cells.

Our spatial biology analysis in cGVHD/BOS directly extends these observations. Early after infusion, CAR19 Tregs predominantly localized to follicular T cell areas, rather than B cell–rich zones. Flow cytometry confirmed that CAR19 Tregs expressed CXCR5 at higher frequencies and MFI than EGFR Tregs, yet this was not sufficient to drive efficient follicular localization. These findings are consistent with prior reports that CXCR5 expression alone is not sufficient for follicular entry, and that additional factors such as chemokine receptor balance, cell activation state, or local microenvironmental cues may influence Treg positioning within secondary lymphoid organs (44-47). It has been suggested that CXCR5-expressing TFRs regulate GC responses at the T–B cell borders to restrict differentiation, entry, and TFH and B cell function (44). CAR19 Tregs localized in extrafollicular areas may exert regulatory control over GC reactions. Migration differences between CAR19 and EGFR Tregs may reflect longer CAR19 Treg engagement with CD19^+^ B cells or chemokine expression changes due to reduced cGVHD injury by CAR19 Tregs. Extrafollicular FoxP3^+^ Treg CTLA-4 expression can suppress GC response initiation, inhibiting T cell proliferation and TFH differentiation (48, 49). This mechanism may contribute to the observed humoral immunity modulation by CAR19 Tregs, even when not physically present within the GC or follicular regions (50). Because measurable B cell depletion was not observed 22 days after infusion and Treg suppression of murine GVHD can occur in the first 2 days after infusion (51), additional spatial imaging time points were not pursued, although longitudinal spatial tracking will be necessary to fully resolve the in vivo CAR19 Treg localization kinetics and functional consequences.

Increased CAR19 Treg contribution to the TFR pool compared with EGFR Tregs was observed (Figure 2D). TFRs play a crucial role in controlling GC responses by limiting TFH and B cell numbers and maintaining self-tolerance (29, 30, 52, 53). TFRs are generated from thymic-derived FoxP3^+^ precursors (29, 30), and, while they share TFH markers (CXCR5, PD-1, BCL6), most TFRs reside at the T–B cell border rather than within GCs themselves (44). In our study, CAR19 Tregs expressing CXCR5, PD-1, and BCL6 may be particularly well positioned to modulate donor T cell activation, TFH differentiation, and B cell response suppression. This is consistent with our observation of decreased TFH frequencies, an effect not observed with EGFR Tregs, and suppression of GCB frequencies.

Our findings, together with other studies, suggest that CAR19 Tregs are more suppressive than EGFR Tregs, likely because of enhanced antigen-specific activation in the presence of B cells. At 22 days after infusion, CAR19 Tregs isolated from cGVHD/BOS spleens exhibited a more suppressive and memory-like phenotype compared with EGFR Tregs. CAR stimulation induced suppressive cytokines and upregulated regulatory markers, consistent with published reports showing that CAR engagement amplifies Treg immunomodulation (32, 34, 42). At baseline, CAR19 Tregs displayed higher canonical suppressive marker expression than EGFR Tregs (Figure 6, B and C) and greater suppression in an in vitro assay with wild-type antigen-presenting cells in the absence of antigen (Figure 6E).

Although our preclinical data suggest that CD8^+^ CAR19 T cells may be insufficient to resolve cGVHD/BOS, CAR19 T cells have shown efficacy in autoimmune disease patients (54). Two separate case reports showed that CAR19 T cells derived from given to patients with recurrent B cell malignancy and active cGVHD of the gastrointestinal tract or lung ameliorated gastrointestinal and sclerodermatous cGVHD (55, 56). Profound and prolonged B cell lymphopenia requiring IgG replacement was seen in both patients. Conventional CAR T cells are known to release proinflammatory cytokines such as TNF-α and IFN-γ, which can amplify local and systemic inflammation and have been implicated in toxicities including cytokine release syndrome (CRS), neurotoxicity, and tissue injury (57–59). In murine models, CD8^+^ CAR19 T cell infusion has been associated with systemic toxicity and rapid lethality due to inflammatory cytokine release (15, 38). Thus, CD8^+^ CAR19 T cells, while depleting B cells, can simultaneously exacerbate or sustain tissue inflammation and fibrosis through cytokine production and bystander immune activation. This dual effect may underlie the lack of improvement in pulmonary function and lymphopenia observed in our preclinical study. It remains to be seen whether CD8^+^ CAR19 T cells in the clinic will prove variably effective. Although CRS (grade I) was noted in only one of two patients (55), the true toxicity risk of CD8^+^ CAR19 T cells infused into cGVHD patients is unknown. As such, our efficacy and lack of toxicity findings in cGVHD/BOS mice given CAR19 Tregs, if extrapolable to the clinic, would represent an advantage over CD8^+^ CAR19 T cells.

Another CAR Treg advantage lies in their ability to home to antigen sites and exert localized, sustained immunosuppression. In an experimental autoimmune encephalomyelitis model, CD4^+^ T cells engineered to express FoxP3 and a CAR targeting myelin oligodendrocyte glycoprotein were able to migrate specifically to the central nervous system, suppress ongoing disease, and maintain protection even after rechallenge, demonstrating durable, antigen-specific regulation (43). Similarly, Boroughs et al. showed that CAR Tregs can accumulate at antigen-rich sites and create a “zone” of immunosuppression, with minimal direct target cell killing, emphasizing the importance of spatial localization for effective immune regulation (32). These findings underscore that, although CAR Tregs are capable of cytolysis in acute or early disease settings (15), their predominant therapeutic effect in established autoimmune and inflammatory diseases appears to result from antigen-driven tissue homing and local immunosuppression. Additionally, as reported by the Rudensky laboratory in a murine lung injury model, amphiregulin secretion by Tregs can have a major direct and non-redundant role in tissue repair and maintenance, distinct from their role in suppression of immune responses and inflammation (60).

While our data indicate that CAR19 Tregs primarily suppress B cell responses through immunomodulation rather than cytolysis in established cGVHD/BOS, the extent to which CAR19 cytolysis can be leveraged to eliminate malignant B cells remains unclear. Recent studies in acute GVHD models suggest that CAR19 Tregs can maintain graft-versus-leukemia responses against CD19^+^ malignant B cells, supporting selective antitumor activity potential (15). In preliminary studies, we have shown that exclusive CAR19 Treg infusion significantly extended survival in mice challenged with hCD19-transduced murine lymphoma cells. Future studies using models of B cell malignancy will be important to determine whether CAR19 Tregs can provide graft-versus-leukemia effects without compromising cGVHD regulation.

In summary, CAR19 Tregs improved pulmonary disease and fibrosis in cGVHD/BOS primarily through potent immunomodulation rather than direct B cell cytolysis, although we cannot exclude the possibility that undetected cytolytic events may have occurred. Suppressive effects were mediated by reshaping of the TFH/TFR axis, reduction of GCB and pathogenic PC differentiation, and lowering of pathogenic IgG2c and collagen deposition. Spatial and phenotypic analyses reveal that while CAR19 Tregs can acquire a follicular phenotype, their localization and function are context dependent and increased CXCR5 expression does not guarantee GC entry. Enhanced suppressive function over EGFR Tregs is likely due to antigen-specific activation and CAR-mediated signaling. These findings provide a foundation for CAR Treg therapy optimization for treating cGVHD and B cell–driven autoimmune diseases, and open new avenues for targeting pathogenic or malignant B cells with precision immunoregulation.

## Methods

### Sex as a biological variable.

Our study used male and female mice. No discernible differences between sexes were identified.

### Mice.

NCI C57BL/6 (B6) and NCI B6-Ly5.1 (CD45.1) female mice were purchased from Charles River Laboratories. B10.BR female mice were purchased from The Jackson Laboratory. B6 Luc/Luc Thy1.1 mice were provided by Robert Negrin (Stanford Medicine, Stanford, California, USA) and bred in our colony. Thomas Tedder (Duke University School of Medicine, Durham, Noth Carolina, USA) provided *hCD19^Tg/Tg^* mice that were bred with B6 mice to generate *hCD19tg^Tg/0^*. B6 FoxP3-GFP knockin mice were provided by Vijay Kuchroo (Harvard University, Boston, Massachusetts, USA) and bred in our colony. All mice were housed in a specific pathogen–free facility and used with University of Minnesota Institutional Animal Care and Use Committee approval.

### Primary T cell and Treg isolation.

Naive CD4^+^ and CD8^+^ T cells (Tcons) were purified from spleens by negative selection using biotin-labeled anti-CD19 (1D3), -B220 (RA3-6B2), -CD11b (M1/70), -CD11c (N418), -NK1.1 (PK136), -CD49b (DX5), -CD25 (PC61.5), -γδ (GL3), and –TER-119 (TER-119) (StemCell Technologies), followed by streptavidin RapidSphere depletion with EasySep magnet (StemCell Technologies). Tregs were purified from spleens and lymph nodes of B6 FoxP3-GFP mice using negative selection but in the absence of anti-CD25 and adding anti-CD8a (53-6.7, StemCell Technologies). CD4^+^CD25^+^ T cells were incubated with PE-labeled anti-CD25 antibody (PC61.5, eBioscience), then incubated with anti-PE microbeads for FoxP3-GFP and anti-APC for EGFR (Miltenyi Biotec) positive selection via magnetic columns (Miltenyi Biotec). Tregs were labeled with anti-CD4 (GK1.5, BioLegend) and fixable viability dye (65-0865-14, Thermo Fisher Scientific) for purification of CD4^+^CD25^hi^GFP^+^ Tregs by fluorescence-activated cell sorting (FACS) on a BD FACSAria II.

### Cell culture.

Purified Tregs were cultured in expansion media with high-glucose and pyruvate DMEM base (11995065, Gibco) supplemented with 10% fetal bovine serum (FBS) (S11595, R&D Systems), 10 mM HEPES (83264, MilliporeSigma), 1× MEM non-essential amino acids (11140050, Gibco), 1× penicillin/streptomycin (15070063, Gibco), 50 μg/mL gentamicin sulfate (30-005-CR, Corning), and 55 μM 2-mercaptoethanol (M6250, MilliporeSigma). T cells were cultured in complete RPMI 1640 with l-glutamine (10-040-CV, Corning) supplemented with 10% heat-inactivated FBS (A5256801, Gibco), 10 mM HEPES (83264, MilliporeSigma), 2 mM GlutaMAX (35050061, Gibco), 1 mM sodium pyruvate (S8636, MilliporeSigma), 1× penicillin/streptomycin (15070063, Gibco), 50 μg/mL gentamicin sulfate (30-005-CR, Corning), and 55 μM 2-mercaptoethanol (M6250, MilliporeSigma).

### Plasmid construction and retrovirus transduction.

The hCD19 construct was subcloned from a lentivirus into an MP71 retroviral vector optimized for T cell expression. For Treg transductions, FACS-purified Tregs were activated with anti-CD3/CD28 Dynabeads (11453D, Gibco) at 3.5 beads/Treg in expansion media with 2,000 IU/mL recombinant human IL-2 (rhIL-2) (Proleukin) and cultured in a humidified incubator at 37°C, 5% CO_2_, on day 0. On day 2, non-tissue-culture-treated 48-well plates were coated with 250 μg/mL RetroNectin (T100B, Takara Bio) overnight at 4°C. On day 3, retrovirus containing tEGFR alone or hCAR19-tEGFR constructs were added to the RetroNectin-coated plates and spinoculated for 2 hours at 2,000*g* (2,980 rpm) at 30°C. Supernatants were aspirated and Tregs added to transduction wells in expansion media with 2,000 IU/mL rhIL-2. Plates were spun at 500*g* (1,500 rpm) for 5 minutes at 30°C. Tregs were cultured at 37°C, 5% CO_2_, with changes of medium every 48 hours until day 7. Transduction efficiency and CAR expression were verified via antibodies for EGFR (anti-EGFR, AY13, BioLegend) and CD19 CAR FMC63 Idiotype (REA1297, Miltenyi Biotec), respectively, by flow cytometry. Tregs were enriched for greater than 90% EGFR frequency and MFI as needed using anti-EGFR in PE or APC (AY13, BioLegend) followed by anti-PE or anti-APC magnetic beads, respectively (Miltenyi Biotec), to be selected via magnetic column. CD8^+^ Tcons were activated with anti-CD3/CD28 Dynabeads at 1 bead/Tcon and cultured in complete RPMI with 300 IU/mL rhIL-2 in a humidified incubator at 37°C, 5% CO_2_. Tcons were retrovirally transduced on culture day 2 following the Treg transduction protocol. On day 4, Tcons were enriched for greater than 90% EGFR frequency and high MFI and replated at 1:1 Dynabead/Tcon ratio in complete RPMI with 300 IU/mL rhIL-2. Tcons were fed every 48 hours until day 7 and transduction purity rechecked before use.

### cGVHD model with CD8^+^ T cell and Treg infusions.

B10.BR recipients were conditioned with 120 mg/kg Cytoxan (Sigma Aldrich) (day –3, –2) and 6.2 Gy total-body irradiation (day –1). Donor BM was harvested from femora, tibiae, and pelves of *hCD19tg^Tg/0^* mice and T cell depleted with anti-CD4 (GK1.5) and anti-CD8 (53-6.7) mAbs and rabbit complement. Splenic T cells were purified from B6 CD45.1 mice using methods described above. Recipients received 1 × 10^7^ TCD BM ± 71.5 × 10^3^ splenic T cells via intravenous tail vein injection. Mice were monitored daily for survival and weighed twice a week until day 30; weights were recorded weekly. Treatment groups received 0.5 × 10^6^ CD8^+^ CAR19 or EGFR T cells or 0.5 × 10^6^ CAR19 or EGFR Tregs on day 28 via tail vein injection.

### Pulmonary function tests.

Mice were anesthetized and ventilated using the flexiVent system (SCIREQ). Pulmonary resistance, elastance, and compliance were measured using flexiWare Software v8.4.1.

### Frozen tissue preparation.

After euthanasia, murine lungs were intratracheally inflated with 70% Tissue-Tek optimal cutting temperature (OCT) compound (4583, Sakura). All tissue blocks (spleen, colon, liver, lung) were flash-frozen in liquid nitrogen and stored at –80°C. Tissues were sectioned in a cryostat at 6 μm thickness onto ProbeOn slides (15-188-51, Fisherbrand).

### Immunofluorescence tissue staining.

For Ig deposition, frozen lung sections were fixed in acetone, stained with goat anti-mouse IgG2c in FITC (ab97254, Abcam), and mounted with Vibrance Antifade Mounting medium with DAPI (H-1800-2, Vector Laboratories). Images were taken on an EVOS microscope and analyzed using R package EBImage via calculation of percentage of pixels most positive in the FITC channel versus the DAPI channel. For GC staining, frozen spleens were fixed in acetone, stained with rhodamine–peanut agglutinin (RL-1072, Vector Laboratories) and anti-CD4–FITC (GK1.5, 11-0042-82, eBioscience), and mounted in Vibrance Antifade Mounting medium with DAPI. Slides were imaged on an EVOS microscope. Voronoi tessellation was used to identify the central peanut agglutinin–positive region of images and quantified for percentage of total pixels in the image.

### Trichrome staining.

Frozen lung sections were stained using a trichrome staining kit (ab150686, Abcam). Slides were mounted with Permount (SP15, Fisher Chemical), imaged using an EVOS microscope, and quantified using EBImage.

### Hematoxylin and eosin staining.

Frozen spleens, lungs, colon, and liver were fixed in acetone and stained with hematoxylin (GH9316, MilliporeSigma) and eosin (HT110116, MilliporeSigma). Slides were mounted with Permount (SP15, Fisher Chemical) and imaged with an EVOS microscope. Stained slides were scored in a blinded fashion using a previously published semiquantitative scoring system. Scores were assigned as 0.5–4.0.

### Flow cytometry.

Cells for B cell staining were incubated with 1:200 Fc block (553142, BD Biosciences) before surface staining. For retained FoxP3 reporter signals, cells were pre-fixed with 2% paraformaldehyde (61) after surface staining before fix/perm and intracellular staining using the Foxp3/Transcription Factor Staining Buffer Set (00-5523-00, Invitrogen), then analyzed on an LSR Fortessa (BD Biosciences). All panels included staining with fixable viability dye (65-0865-14, Thermo Fisher Scientific). Fluorochrome-conjugated mAbs included anti-CD4 (RM4-5, BioLegend), anti-EGFR (AY13, BioLegend), anti–idiotype CD19 CAR FMC63 (REA1297, Miltenyi Biotec), anti–mouse CD19 (eBio1D3, eBioscience), anti-hCD19 (HIB19, BioLegend), anti-FoxP3 (FJK-16s, eBioscience), anti-CXCR5 (SPCL5, eBioscience), anti–PD-1 (RPM1-30, eBioscience), anti-CD95 (SA367H8, BioLegend), anti-GL7 (GL7, eBioscience), anti-BCL6 (BCL-DWN, eBioscience), anti-B220 (RA3-6B2, eBioscience), anti-CD138 (281-2, BioLegend), anti-CD107a (1D4B, BioLegend), anti-FasL (MFL3, eBioscience), anti-perforin (eBioOMAK-D, eBioscience), anti–granzyme A (3G8.5, eBioscience), anti–granzyme B (NGZB, eBioscience), anti–TNF-α (MP6-XT22, eBioscience), anti–IFN-γ (XMG1.2, eBioscience), anti–IL-10 (JES5-16E3, eBioscience), anti-Tigit (GIGD7, eBioscience), anti–CTLA-4 (UC10-4B9, eBioscience), anti-CD127 (A7R34, eBioscience), anti-LAG3 (C9B7W, eBioscience), and anti-KLRG1 (2F1, eBioscience).

### In vitro Treg suppression assay.

TCD splenocytes were isolated from B6 Luc/Luc Thy1.1 or hCD19tg F1 mice. CD25-depleted T cells were isolated from B6 Luc/Luc Thy1.1 mice and stained with 5 μM CellTrace CFSE (C34570, Invitrogen) for 5 minutes at room temperature in the dark, then plated at 1:1 Tcons/TCD splenocytes with 0.25 μg/mL anti-CD3ε (16-0031-82, Thermo Fisher Scientific) to stimulate T cell proliferation.

### Flow killing assays.

B cells were isolated from naive B6 or *hCD19tg^Tg/0^* or day 28 cGVHD transplanted mice. B cells were added to Tregs and CD8^+^ T cells at 5:1 effector/target ratios for 48 hours. B cells from naive mice were activated during the assay with 5 μg/mL anti-IgM (16-5092-85, Thermo Fisher Scientific) and 100 ng/mL recombinant human BAFF (310-13, PeproTech). Killing was measured through flow cytometry of targets stained with Fixable Viability dye (65-0865-18, Invitrogen). For assays using serum, peripheral blood was collected from day 28 BM-only or cGVHD transplanted mice, incubated at room temperature for 30 minutes, and then centrifuged at 2,000*g* for 10 minutes. Serum was carefully removed and stored at –80°C until use. Serum was added to culture at 1:10 dilution. For Transwell (3381, Corning) killing assays, B cells isolated from naive *hCD19tg^Tg/0^* mice were added to the bottom wells and activated with 5 μg/mL anti-IgM (16-5092-85, Thermo Fisher Scientific) and 100 ng/mL recombinant human BAFF (310-13, PeproTech). CAR19 or EGFR Tregs were added to the top insert or bottom wells at 5:1 effector/target ratios for 48 hours. B cells from the bottom wells were harvested for flow cytometry analysis of killing through Fixable Viability dye (65-0865-18, Invitrogen) staining.

### In vivo killing model.

As published (15, 38), 3 × 10^6^ effectors were adoptively transferred via tail vein injection into *hCD19tg^Tg/0^* recipients that received lymphodepleting Cytoxan (300 mg/kg) on day –1. Target tissues were harvested for analysis of day 5 after effector infusion.

### CellScape.

Spleen sections were flash-frozen in OCT without prior fixation. Coverslips included in the CellScape Tissue Chip Kit (PRSM-CHP-TISSUE, Canopy Biosciences) were coated with poly-l-lysine (P8920, MilliporeSigma) for 5 minutes at room temperature and dried. Spleen tissues were sectioned at 7 μm thickness onto the coated coverslips and set overnight at –80°C. On the day of run, tissues were pre-fixed in fresh 4% paraformaldehyde (15710, Electron Microscopy Sciences) at room temperature for 5 minutes, then washed with cold 1× PBS for 5 minutes on ice. Tissues were fixed in acetone (5 minutes, 4°C), 90% ethanol (3 minutes, 4°C), 70% ethanol (3 minutes, 4°C), and 1× PBS buffer (5 minutes, 4°C). 1× perm buffer from the Foxp3/Transcription Factor Staining Buffer Set (Invitrogen, 00-5523-00) was used as working solution for intracellular stain cycles. Fluorochrome-conjugated mAbs included anti-GFP (600-102-215, Rockland), anti-hCD19 (HIB19, BioLegend), anti–PD-1 (29F.1A12, BioLegend), and anti-CXCR5 (2G8, BD Biosciences). Peanut agglutinin in fluorescein (FL-1071, Vector Laboratories) was used for GC staining. Coverslips were loaded onto the tissue chips and images acquired on Bruker’s Canopy Spatial Biology platform.

### Spatial proteomics analysis.

OME-TIFF files output from CellScape platform underwent nuclear segmentation using CellPose v4 (CP-SAM model) (62). To assist in background removal, individual channels were pre-processed by tile normalization and application of standard corrections to brightness and contrast (Supplemental Methods). A foreground tissue mask was generated from the nuclear channel (DNA-BV421) using Otsu thresholding on a down-sampled image and per-cell marker intensities computed on inside mask regions. Resulting data were loaded into R as a SpatialExperiment object (63). To remove extreme outliers, each channel was clipped to its 99th percentile followed by per-channel normalization. Unsupervised clustering and manual annotation were performed via FuseSOM to identify cell types (64). Spatial colocalization between cell types was quantified via spicyR (65) using the L-function, a transformation of Ripley’s K-function. Spatial domains were identified using the lisaClust package (66), which computes the local indicators of spatial association (LISA) functions for each cell at multiple radii to assemble regions defined by coordinated cell type arrangements. Resulting regions were manually annotated based on cell type distribution patterns. Spatial domain boundary interfaces were determined via Voronoi tessellation computed using the deldir package.

### Statistics.

Data are presented as mean ± SEM. Statistical analyses were performed using unpaired 2-tailed *t* test, 1-way ANOVA with Tukey’s correction for multiple comparisons, or 2-way ANOVA with Šidák’s correction for multiple comparisons through GraphPad Prism version 9 software. *P* values less than 0.05 were considered statistically significant.

### Study approval.

Animal studies were conducted in accordance with a protocol reviewed and approved by the Institutional Animal Care and Use Committee of the University of Minnesota (2403-41945A).

### Data availability.

All supporting data values are compiled in the Supporting Data Values file. Code used for CellScape data analysis is available at https://github.com/Anorinoth/P022-SJ-2024-CellScape Raw image data are available in the BioImage Archive at accession number S-BIAD3501.

## Author contributions

SJ designed and performed experiments, analyzed results, and wrote the manuscript. MCZ analyzed results and edited the manuscript. CMH designed and performed experiments, analyzed results, and edited the manuscript. CRH, SBW, and JHL contributed to experimental design and data collection, discussed results, and edited the manuscript. YP and SH generated retroviruses. MR assisted in tissue cutting for spatial proteomics. APM scored histopathology sections. AS, YP, EK, YZ, RAM, and PSMB contributed to data collection. APM, KLH, CRE, WJM, GRH, PTS, LSK, CAP, and JT discussed experiments, results, and conclusions. BRB designed experiments, reviewed data, and assisted in manuscript preparation. SJ, MCZ, and CMH contributed equally to this work. THe order of co–first authors was determined by first involvement in the project. SJ and CMH each designed, completed and analyzed experiments. MCZ performed all spatial proteomic analyses that make up figures 5 and 6. All three authors contributed equally to the writing of the manuscript.

## Conflict of interest

BRB reports consulting fees from BlueRock Therapeutics, Sanofi, Legend Biotech, GentiBio Inc., Incyte Corp., Atara Biotherapeutics, Affyxell Co., and Therakos Inc. GRH has consulted for Generon Corp., NapaJen Pharma, iTeos Therapeutics, Commonwealth Serum Laboratories, Cynata Therapeutics, Neoleukin Therapeutics, and Incyte Pharma and has received research funding from Compass Therapeutics, Syndax Pharmaceuticals, Applied Molecular Transport, Serplus Technology, Heat Biologics, Laevoroc Oncology, iTeos Therapeutics, Genentech, Incyte Pharma, and Commonwealth Serum Laboratories. LSK is on the scientific advisory board of hiFiBiO and reports research funding from Magenta Therapeutics, Tessera Therapeutics, Novartis, Emmanuel Merck, Darmstadt Serono, Gilead Pharmaceuticals, and Regeneron Pharmaceuticals, consulting fees from Vertex, and grants and personal fees from Bristol Myers Squibb. LSK reports a conflict of interest with Bristol Myers Squibb, which is managed under an agreement with the Harvard Medical School.

## Funding support

This work is the result of NIH funding, in whole or in part, and is subject to the NIH Public Access Policy. Through acceptance of this federal funding, the NIH has been given a right to make the work publicly available in PubMed Central.

- NIH R37 AI34495, R01 HL11879, P01 HL158505, and P01 AI056299 (to BRB); R01 HL148164 and P01 CA018029 (to GRH); R01 CA263090 (to CAP); and R01 HL095791, P01 HL158504, and U19 AI051731 (to LSK).
- Children’s Cancer Research Fund (to MCZ).

## Supplementary Material

Supplemental data

Supporting data values

## Version 1 07/14/2026

In-Press Preview

## Version 2 08/24/2026

Electronic publication
