# Supplementary material for: CAR19 Tregs treat murine chronic graft-versus-host disease through immune suppression without measurable B cell cytolysis
Source: JCI Insight. 2026 Jul 14;11(16):e196197. doi: 10.1172/jci.insight.196197 (PMC13502169; doi:10.1172/jci.insight.196197)
Supplement: Supplementary file 1 [file jciinsight-11-196197-s108.pdf]

Supplemental Methods: Image Preprocessing, Cell Segmentation, and Quality Control

Spectral Bleed through Correction:

Raw 30-channel pyramidal OME-TIFF images acquired on the CellScape multiplex immunofluorescence platform were preprocessed to correct for spectral bleed through of the CD19-FITC signal into the FoxP3-GFP channel. Individual channel images were extracted from the multiplexed OME-TIFF using the Bio-Formats command-line tool with 1024 x 1024 pixel tiling and LZW compression. A bleed through-corrected FoxP3 channel was generated by applying a uniform averaging filter (15 x 15 pixel kernel) to the extracted CD19-FITC image to capture spatially diffuse peripheral bleed through, scaling the blurred image by a factor of 5.0, and subtracting it from the GFP-FITC image with negative values clipped to zero. In the third phase, all original channels and the corrected FoxP3 channel were reassembled into a single pyramidal OME-TIFF.

Foreground Masking and Nuclear Segmentation

A binary tissue mask was generated from the nuclear channel (DNA-Brilliant Violet 421) to exclude background regions from analysis. The nuclear image was down-sampled by a factor of 8, and an intensity threshold was determined using Otsu's method applied to nonzero pixels. The resulting binary mask was dilated using a disk-shaped structuring element (radius = 10 pixels), and small holes were removed (area threshold = 500 pixels). The mask was then up-sampled to the original image resolution. Nuclear segmentation was performed using Cellpose v4 with the CP-SAM model with GPU acceleration. The nuclear channel (DNA-Brilliant Violet 421) was used as input. Cellpose was configured with auto-detected nuclear diameter, a cell probability threshold of 0.0, a minimum cell size of 15 pixels, tile normalization block size of 100, flow threshold of 0.4, test-time augmentation was disabled, and a tile overlap of 0.1 was used.

Channel Enhancement and Quantification and Normalization

For enhanced OME-TIFF export, each channel was processed through an adaptive enhancement pipeline consisting of three steps: (1) percentile normalization to a [0, 1] range using the 0.5th and 99.5th percentiles of nonzero pixels; (2) outlier masking, in which pixel values exceeding the 98th percentile were replaced with the channel median; (3) blending of the original image (70%) with a contrast-limited adaptive histogram equalization (CLAHE) image (30%, clip limit = 0.005). Resultant per channel quantifications were combined and imported into R as a 'SpatialExperiment' object. Cell-level marker intensities were normalized by; per-marker outlier trimming at the 99th percentile, mean-centering, batch correction.

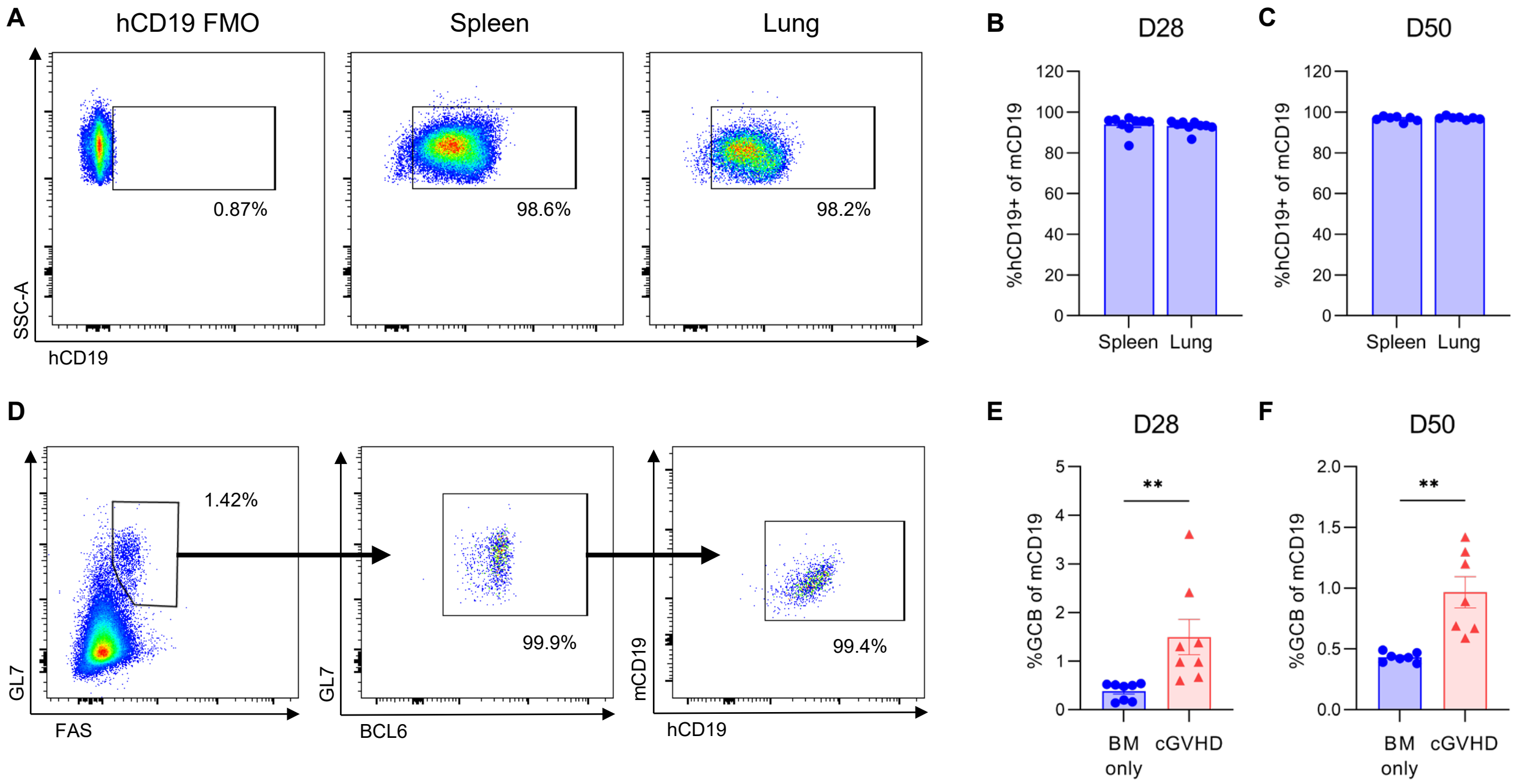

Supplementary Figure 1. hCD19tg$^{Tg/0}$ BM grafts in murine cGVHD/BOS model result in high hCD19 expression in mature B-cells and splenic germinal center B-cells by Day 50 post-transplant. (A) Representative flow cytometry plots of hCD19 expression on live mCD19+ lymphocytes from the spleen [middle] and lung [right] of cGVHD mice on Day 28 post-transplant. Frequency of hCD19 expression on mCD19+ live lymphocytes in target tissues on (B) Day 28 vs (C) Day 50 post-transplant. (D) Gating strategy for GCBs (GL7+FAS+BCL6+) gated on live mCD19+ cells. Frequency of GCB cells in control BM-only vs cGVHD mice on (E) Day 28 and (F) Day 50 post-transplant. Data representative of 2 independent experiments. Statistics shown are results of unpaired Student's T-tests. *p < 0.05, **p < 0.01, ***p < 0.001, ****p < 0.0001. GCB: germinal center B-cells

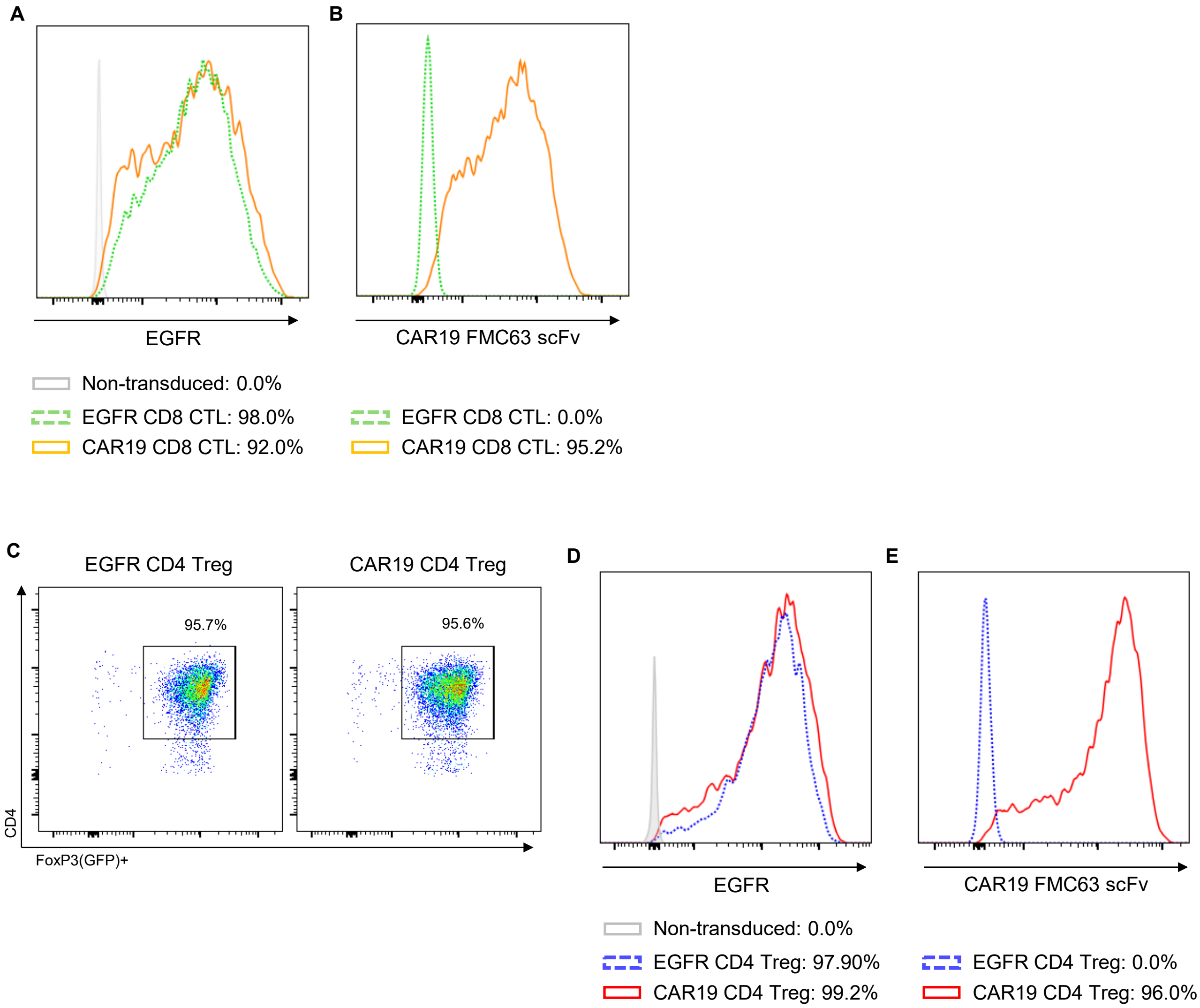

Supplementary Figure 2. Purity of murine CAR19 or EGFR CD8 CTLs and CD4 Tregs. Representative histogram plots of (A) truncated human epidermal growth factor receptor (EGFR) and (B) CAR19 FMC63 scFv in non-transduced, EGFR, and CAR19 CD8 CTLs on Day 7 of culture prior to experimental use. (C) Representative flow cytometry plots of CD4+FoxP3(GFP)+ purity gated on live lymphocytes of EGFR and CAR19 Tregs on Day 7 of culture prior to experimental use. Representative histogram plots of (D) truncated human epidermal growth factor receptor (EGFR) and (E) CAR19 FMC63 scFv in non-transduced, EGFR, and CAR19 CD4 Tregs.

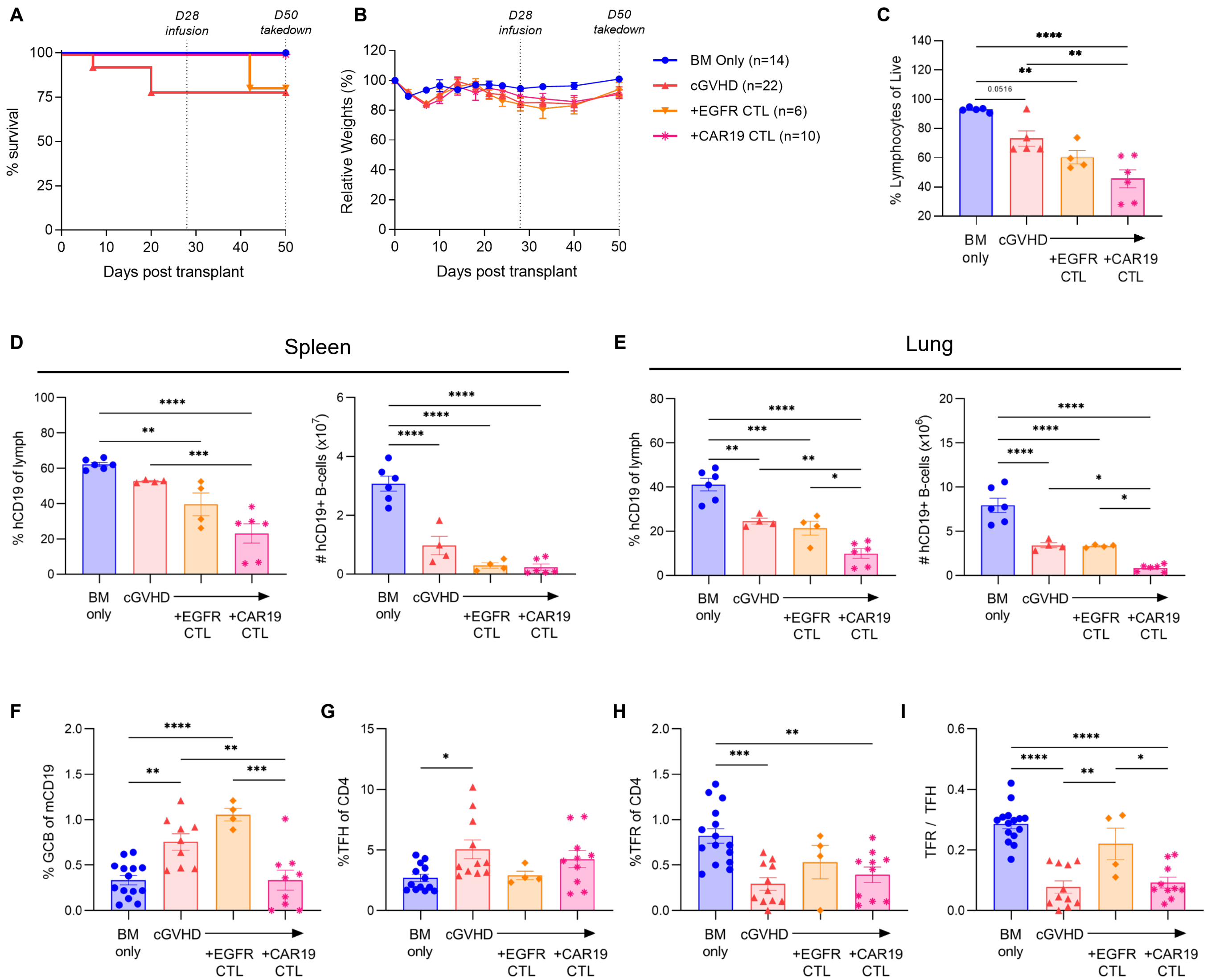

Supplementary Figure 3. Day 28 infusion of CAR19 CTLs do not treat murine cGVHD/BOS despite depletion of bulk B-cells and germinal center B-cells. (A) Survival curve and (B) relative weights of cGVHD mice treated with Day 28 infusion of EGFR or CAR19 CD8 CTLs. All flow analyses was performed on Day 50 post-transplant. (C) Transplanted mice were analyzed for splenic lymphocytes of live. (D) Spleens and (E) lungs were analyzed for frequencies and counts of hCD19+ B-cells of lymphocytes. Spleens were analyzed for frequencies of (F) GCB of mCD19, (G) TFH of CD4, (H) TFR of CD4, and (I) TFR/TFH ratios. Data pooled from two independent experiments. Statistics shown are results of one-way ANOVA with Tukey correction for multiple comparisons. *p < 0.05, **p < 0.01, ***p < 0.001, ****p < 0.0001.

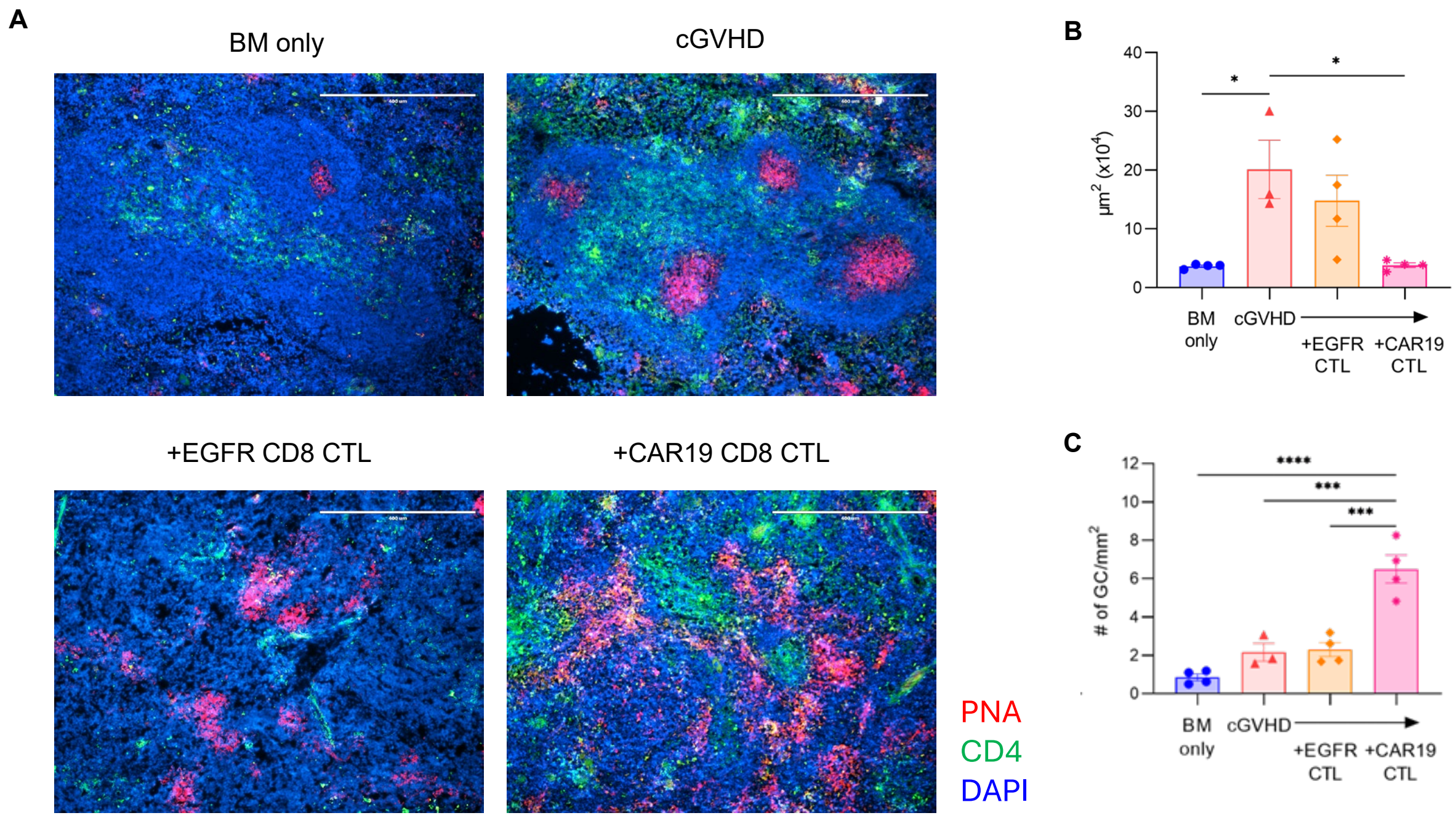

Supplementary Figure 4. Day 28 infusion of CAR19 CTLs result in incomplete germinal center formations. (A) Representative 100x images of spleen sections stained for germinal centers with PNA (red), CD4 (green) and DAPI (blue) and quantified for (B) germinal center size. (C) Representative 100x images of spleen sections stained for germinal centers and quantified for (D) frequency of germinal centers per mm^2. Statistics shown are results of one-way ANOVA with Tukey correction for multiple comparisons. *p < 0.05, **p < 0.01, ***p < 0.001, ****p < 0.0001.

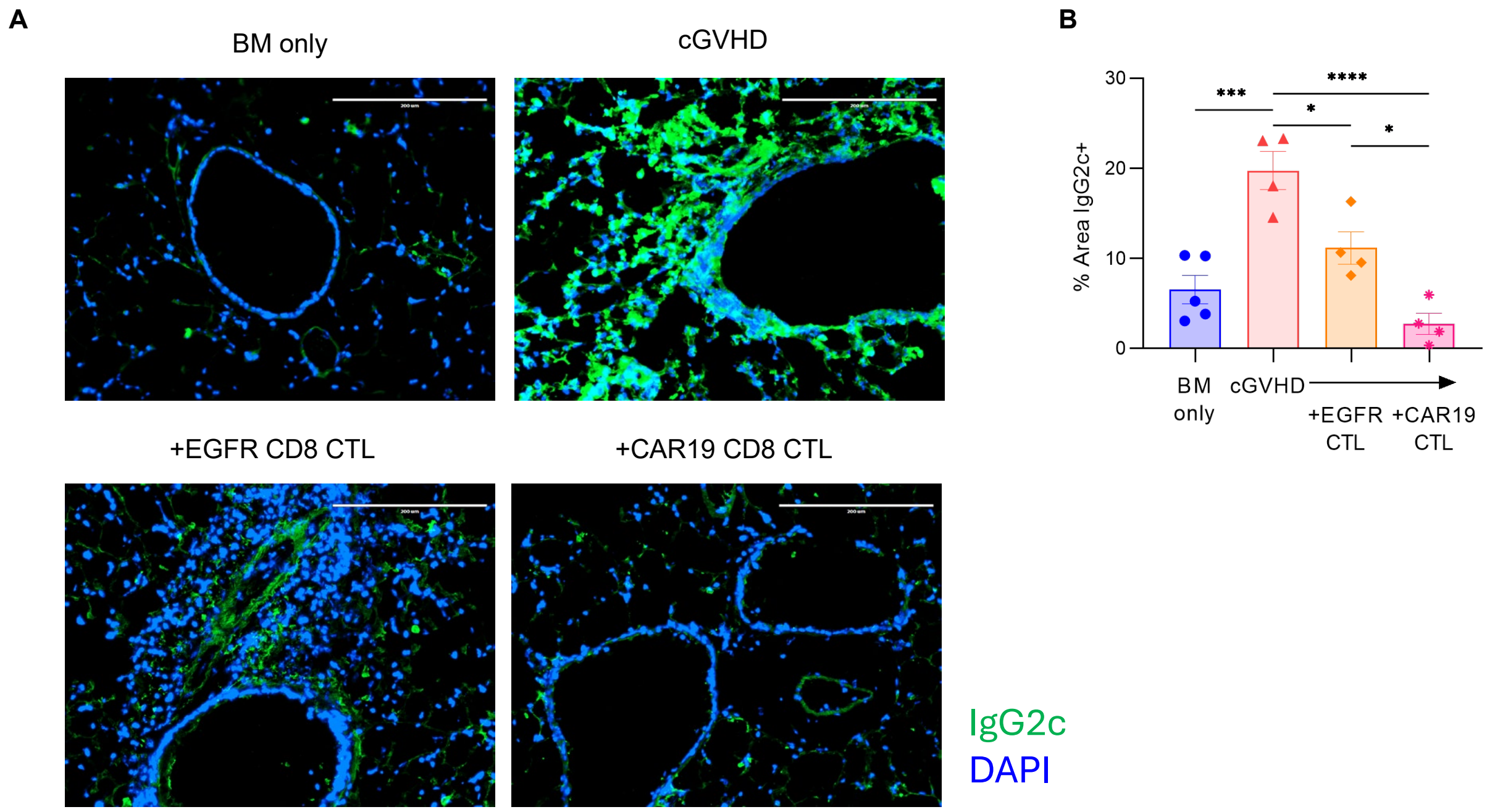

Supplementary Figure 5. Day 28 infusion of CAR19 CTLs reduces pathogenic IgG2c deposition in cGVHD/BOS lungs. (A) Representative 200x images of lung sections stained for antibody deposition with IgG2c (green) and DAPI (blue). (B) Quantification of IgG2c deposition images for % area IgG2c+. Statistics shown are results of one-way ANOVA with Tukey correction for multiple comparisons. $^*p < 0.05$, $^{**}p < 0.01$, $^{***}p < 0.001$, $^{****}p < 0.0001$.

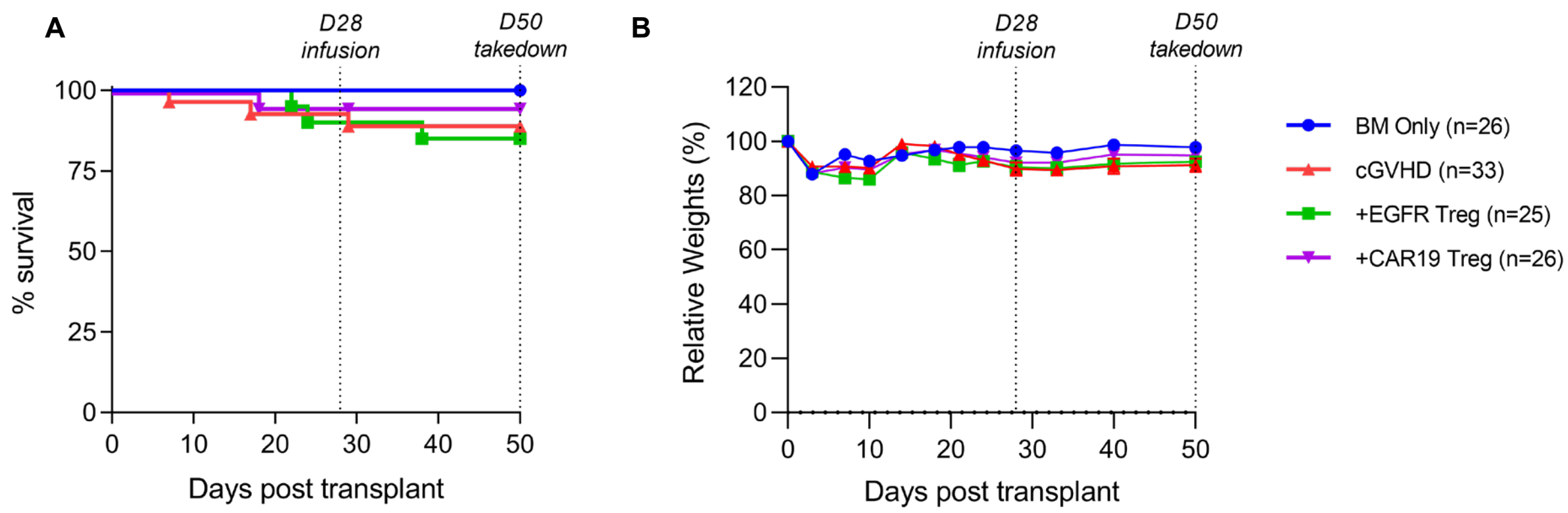

Supplementary Figure 6. Day 28 CAR19 Treg infusions do not impact survival or relative weights during cGVHD/BOS. (A) Survival curve and (B) relative weights of cGVHD mice treated with Day 28 infusion of EGFR or CAR19 Tregs. Data pooled from four independent experiments.

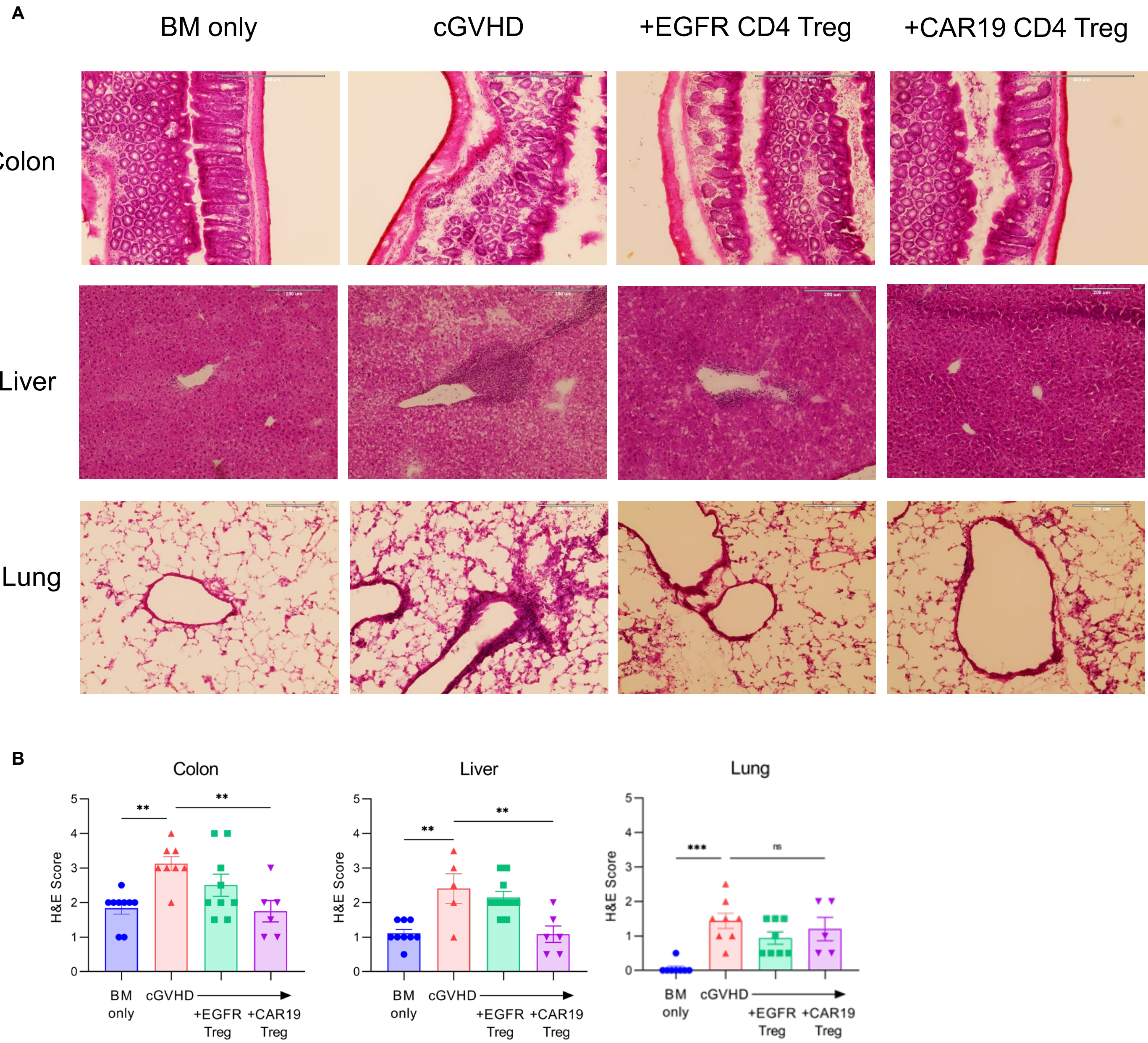

Supplementary Figure 7. Day 28 CAR19 Treg infusions reduce murine cGVHD/BOS disease. (A) Representative 200x images of cryopreserved tissue sections stained with hematoxylin and eosin for blinded histopathology scoring. (B) H&E pathology scores for each tissue section shown. Statistics shown are results of one-way ANOVA with Tukey correction for multiple comparisons. $*p < 0.05$, $**p < 0.01$, $***p < 0.001$, $****p < 0.0001$.

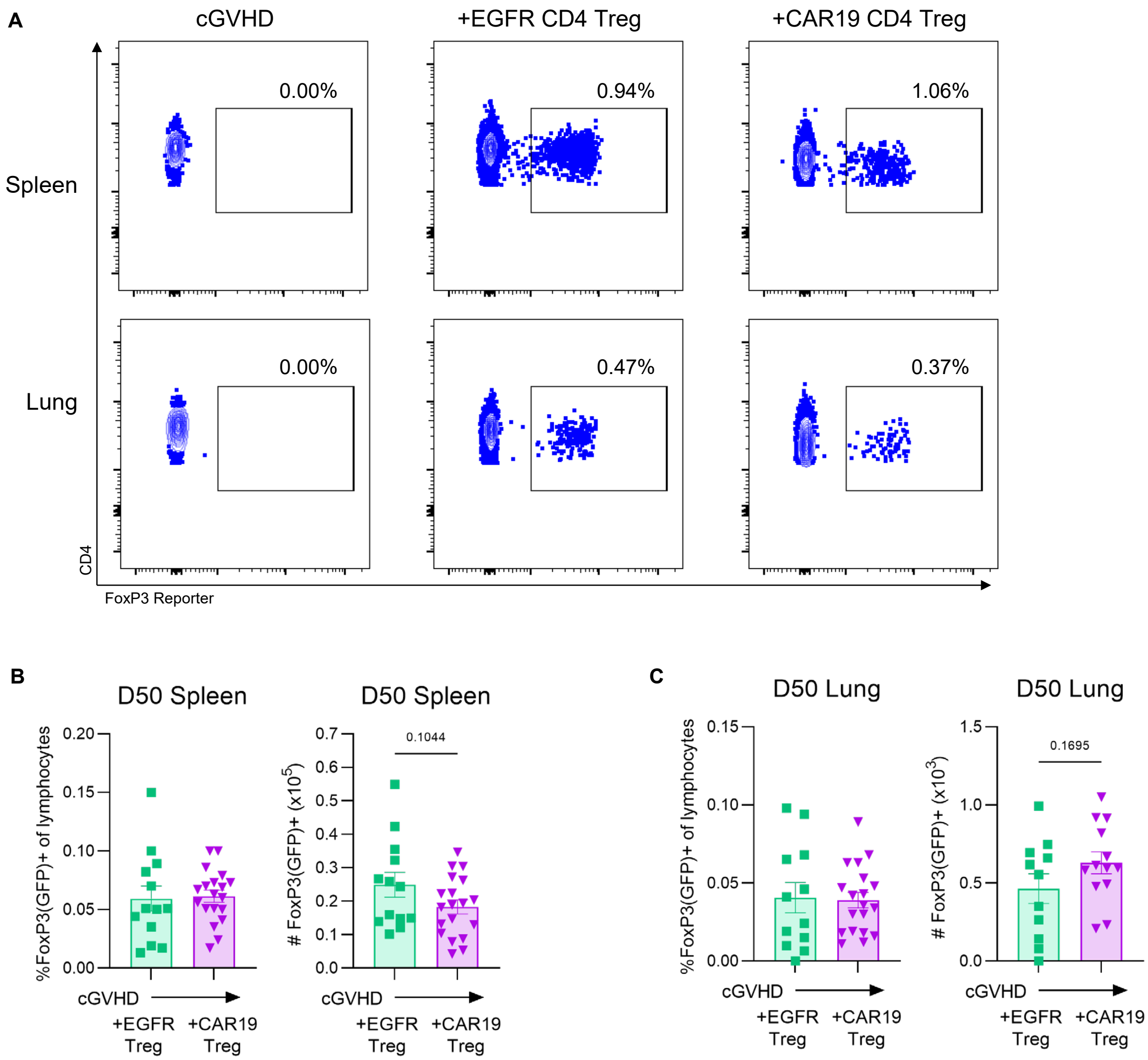

Supplementary Figure 8. Adoptively transferred CAR19 and EGFR Tregs are found at similar frequencies and counts in Day 50 post-transplant target tissues. (A) Representative flow cytometry plots of infused GFP+ CAR19 or EGFR Tregs in spleen and lungs tissues gated on live lymphocytes. Plots display control untreated cGVHD mice compared to Treg-treated groups. Frequencies of FoxP3(GFP)+ cells of lymphocytes [left] and total counts [right] in the (B) spleen and (C) lung per lower right lobe at Day 50 post-transplant. Statistics shown are results of unpaired Student's T-Tests. *$p < 0.05$, **$p < 0.01$, ***$p < 0.001$, ****$p < 0.0001$.

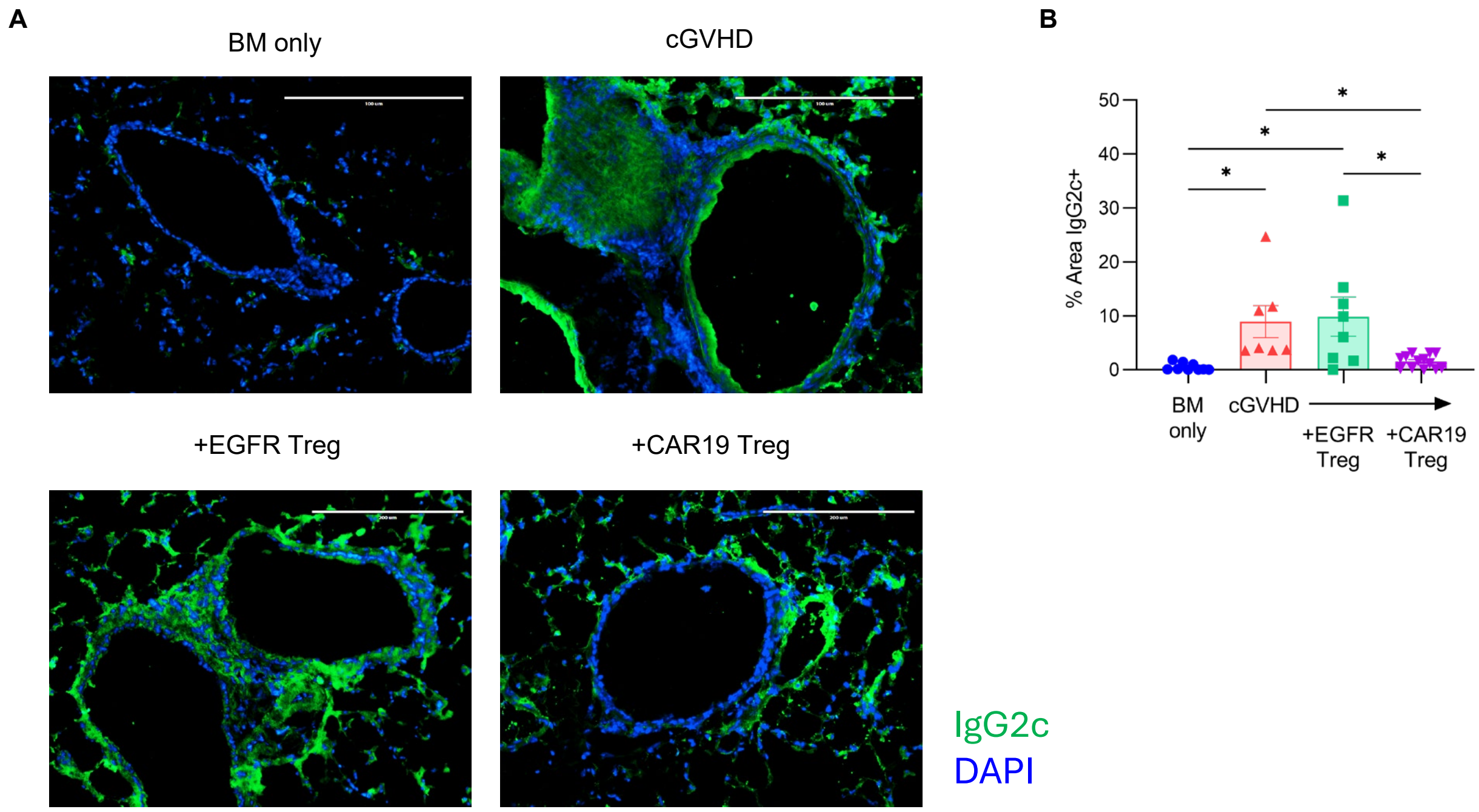

Supplementary Figure 9. Day 28 CAR19 Treg infusion decrease pathogenic IgG2c deposition cGVHD/BOS lungs. (A) Representative 200x images of lung sections stained for antibody deposition with IgG2c (green) and DAPI (blue). (B) Quantification of IgG2c deposition images for % area IgG2c+. Statistics shown are results of one-way ANOVA with Tukey correction for multiple comparisons. *$p < 0.05$, **$p < 0.01$, ***$p < 0.001$, ****$p < 0.0001$.

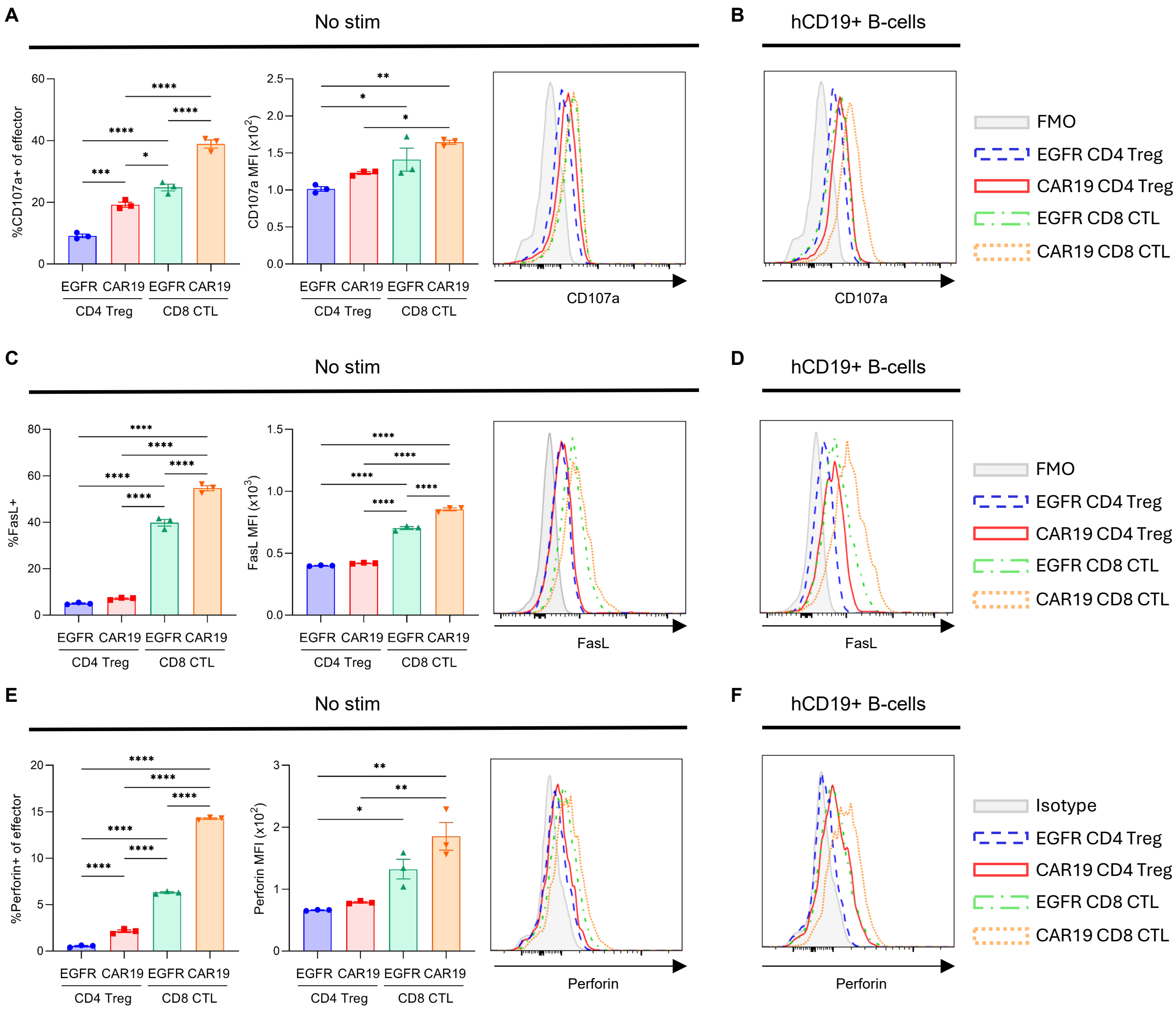

Supplementary Figure 10. Expression of cytolytic markers at baseline and post co-culture of hCD19tg$^{Tg/0}$ B-cells. (A) Frequency and MFI with representative histograms of CD107a at 5 hours of no stim or hCD19+ co-culture at 1 Effector: 5 Target. (B) Frequency and MFI with representative histograms of FasL at 48 hours of no stim or hCD19+ co-culture at 1 Effector: 5 Target. (C) Frequency and MFI with representative histograms of Perforin at 24 hours of no stim or hCD19+ co-culture at 1 Effector: 5 Target. Data are representative of 3 independent experiments. Statistics shown are results of one-way ANOVA with Tukey correction for multiple comparisons. *p < 0.05, **p < 0.01, ***p < 0.001, ****p < 0.0001.

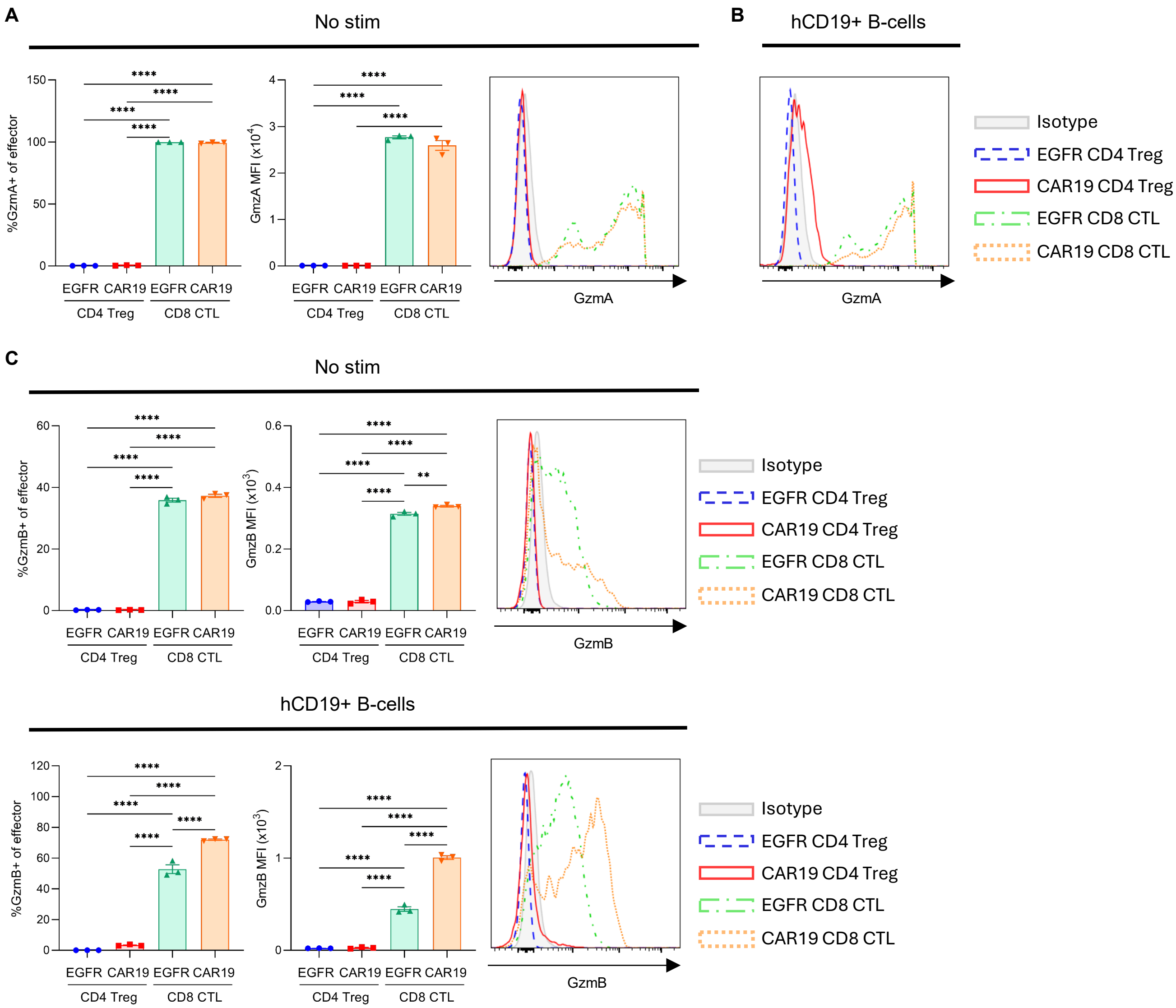

Supplementary Figure 11. Expression of cytolytic markers at baseline and post co-culture of hCD19tg$^{Tg/0}$ B-cells. Frequency and MFI with representative histograms of (A) Granzyme A or (B) Granzyme B at 48 hours of no stim or hCD19+ co-culture at 1 Effector: 5 Target. Data are representative of 3 independent experiments. Statistics shown are results of one-way ANOVA with Tukey correction for multiple comparisons. $^*p < 0.05$, $^{**}p < 0.01$, $^{***}p < 0.001$, $^{****}p < 0.0001$.

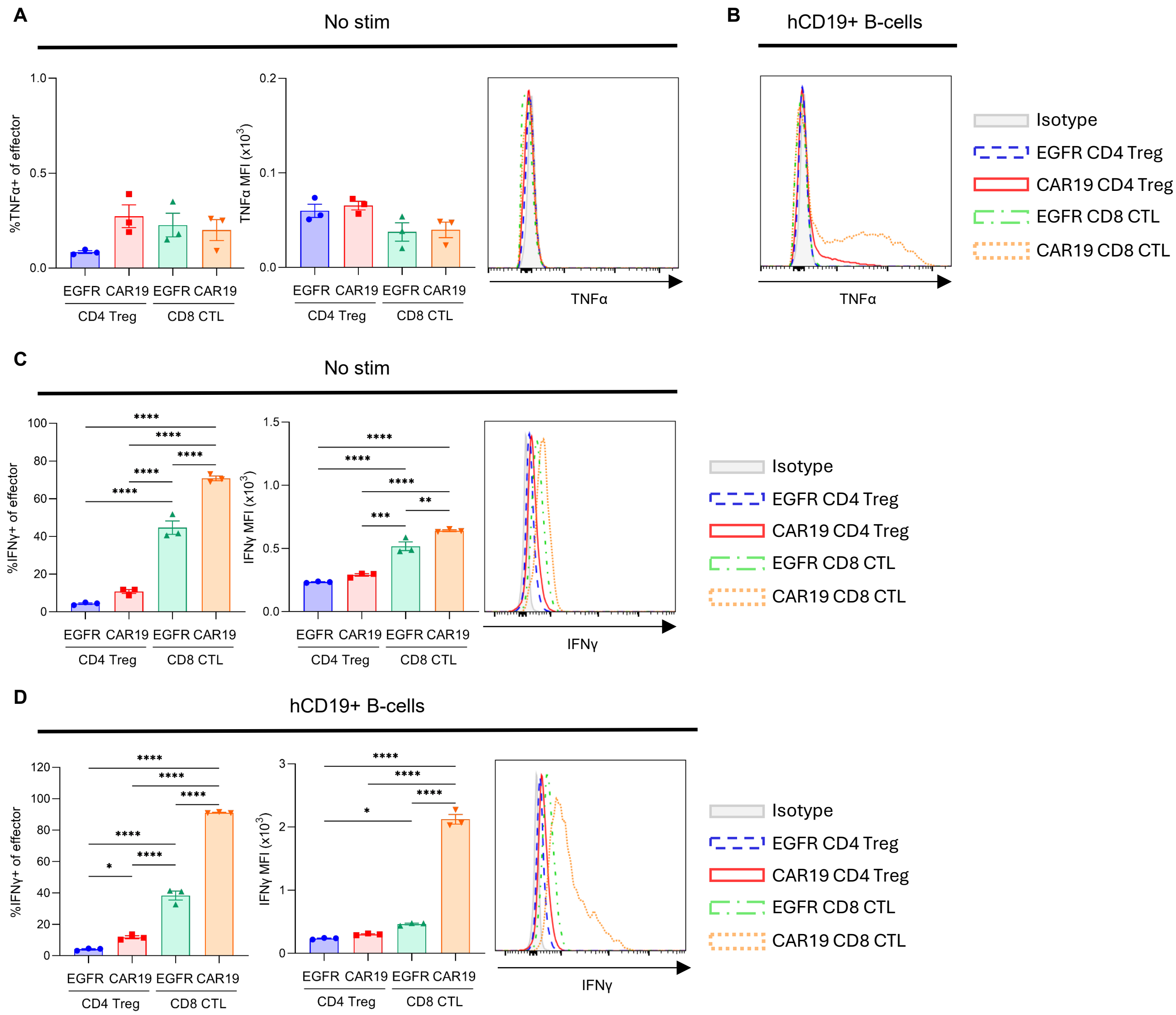

Supplementary Figure 12. Expression of cytolytic markers at baseline and post co-culture of hCD19tg$^{Tg/0}$ B-cells. Frequency and MFI with representative histograms of (A) TNFα or (B) IFNγ at 24 hours of no stim or hCD19+ co-culture at 1 Effector: 5 Target. Data are representative of 3 independent experiments. Statistics shown are results of one-way ANOVA with Tukey correction for multiple comparisons. *$p < 0.05$, **$p < 0.01$, ***$p < 0.001$, ****$p < 0.0001$.

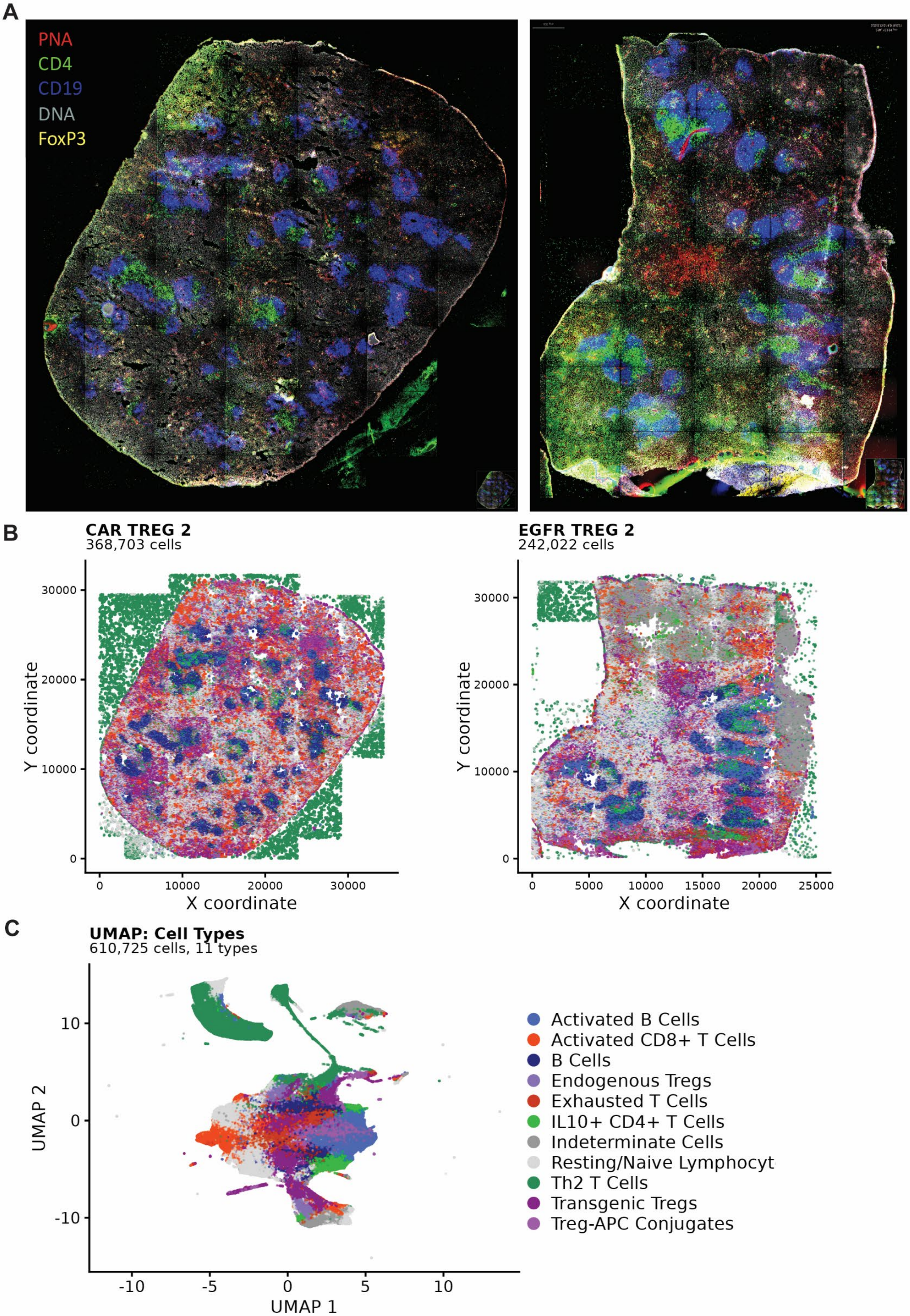

Supplemental Figure 13. Cell segmentation and annotation of multiplex immunofluorescence images. (A) Multiplex immunofluorescence images of spleen tissue taken 5 days after infusion of either CAR19 Tregs (left) or EGFR Tregs (right). Channels shown include PNA (red), CD4 (green), CD19 (blue), DAPI (grey), and FoxP3 (yellow). Images shown are after standard corrections to brightness and contrast to enhance and normalize visualization prior to cell segmentation. See supplemental methods for details of image preprocessing. (B) Spatial reconstruction of tissue using cell type annotations. By-cell channel quantifications were clustered using FuseSOM, and resultant clusters were manually annotated based channel intensity patterns. (C) UMAP of cells across both images after all cell annotation is concluded.

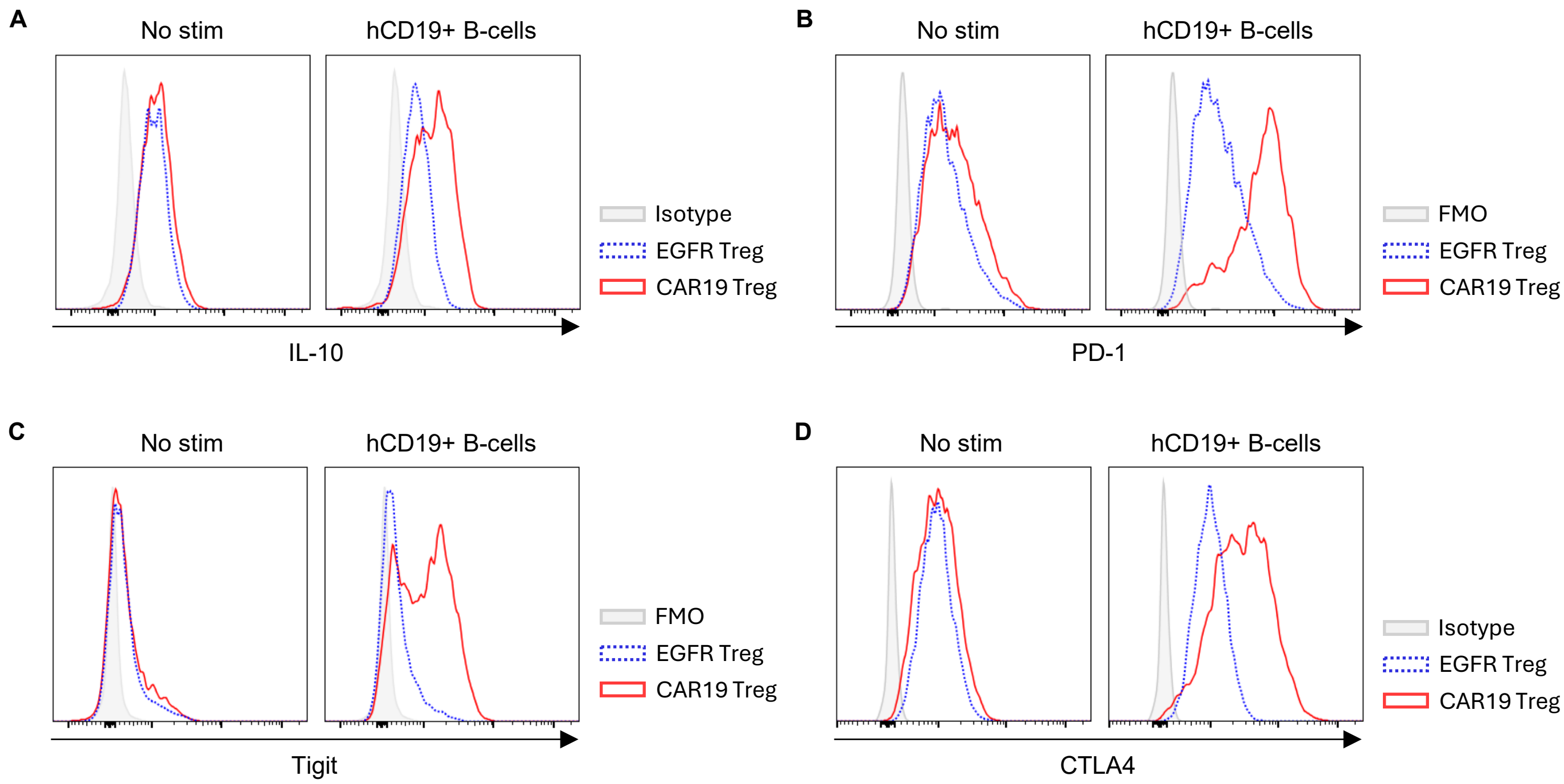

Supplementary Figure 14. CAR19 Tregs have increased expression of suppressive markers at baseline and post antigen-specific activation by hCD19tg$^{Tg/0}$ B-cells. Representative histograms at 48 hours no stim [left] and co-culture with hCD19+ B-cells of (A) IL-10, (B) PD-1, (C) Tigit, (D) CTLA4.

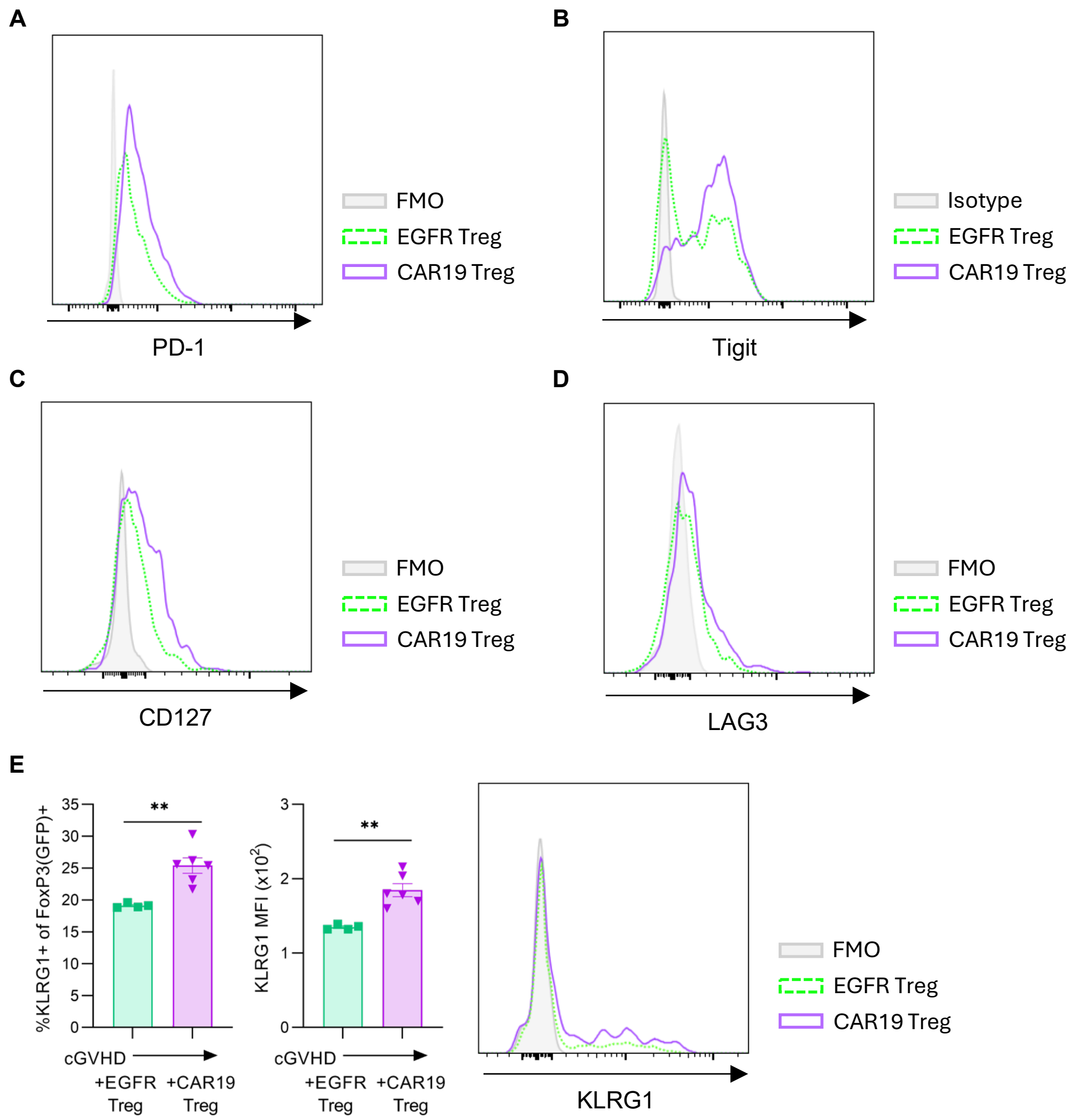

Supplementary Figure 15. *In vivo* Day 50 CAR19 Tregs have a more suppressive and memory-like phenotype. Representative histograms of (A) PD-1, (B) Tigit, (C) CD127, (D) LAG3. (E) Frequency, MFI, and representative histogram of KLRG1. Data are representative of 3 independent experiments. Statistics shown are results of unpaired Student's T-Tests. *p < 0.05, **p < 0.01, ***p < 0.001, ****p < 0.0001. MFI: mean fluorescence intensity.
